# Burden and Projections of Premenopausal and Postmenopausal Breast Cancer Worldwide: A Comprehensive Analysis of GLOBOCAN 2022 Data

**DOI:** 10.1155/tbj/1290471

**Published:** 2026-11-04

**Authors:** Rongbo Ding, Hongyan Jia

**Affiliations:** ^1^ Department of Breast Surgery Heping Hospital Affiliated to Changzhi Medical College Changzhi Shanxi China; ^2^ Department of Breast Surgery First Hospital of Shanxi Medical University Taiyuan Shanxi China sxmu.edu.cn

**Keywords:** breast cancer, GLOBOCAN 2022, menopausal status, projection, temporal trend

## Abstract

**Objectives:**

Breast cancer is the most diagnosed cancer among women worldwide, yet important epidemiological differences exist between premenopausal and postmenopausal disease. Updated global estimates stratified by menopausal status are limited. This study aimed to quantify the global, regional, and national burden of premenopausal and postmenopausal breast cancer in 2022 and to project future trends through 2050.

**Methods:**

Breast cancer incidence and mortality data for 2022 were obtained from the International Agency for Research on Cancer GLOBOCAN database. Women aged 15–49 years were classified as premenopausal and those aged ≥ 50 years as postmenopausal. Age‐standardized incidence rates (ASIRs), age‐standardized mortality rates (ASMRs), cumulative risk, and mortality‐to‐incidence ratios (MIRs) were estimated for 185 countries. Countries were further stratified by the Human Development Index (HDI) published by the United Nations Development Programme. Temporal trends were assessed using estimated annual percentage change (EAPC), and projections to 2050 were derived using the World Health Organization Cancer Tomorrow tool.

**Results:**

In 2022, an estimated 2,296,607 new breast cancer cases and 666,061 deaths occurred globally. Premenopausal women accounted for 668,417 cases (29.1%) and 141,338 deaths (21.2%), while postmenopausal women accounted for 1,628,190 cases (70.9%) and 524,723 deaths (78.8%). Global ASIRs were 32.7 and 153.9 per 100,000 for premenopausal and postmenopausal women, respectively. Incidence rates were highest in very high‐HDI countries, whereas mortality and MIR were markedly higher in low‐HDI countries. Africa showed the highest MIRs for both premenopausal (40.3%) and postmenopausal (51.8%) breast cancer. HDI was positively correlated with cumulative incidence risk but strongly inversely correlated with MIR. By 2050, global cases are projected to increase to 795,908 for premenopausal and 2,756,889 for postmenopausal breast cancer.

**Conclusions:**

Substantial global disparities exist in breast cancer burden and outcomes. While high‐income countries experience higher incidence, low‐resource settings bear disproportionately higher mortality. Strengthening early detection, treatment access, and cancer control systems is essential to reduce global inequities and address the growing burden of breast cancer.

## 1. Introduction

Breast cancer is the most commonly diagnosed malignancy among women and the leading cause of cancer‐related death worldwide, representing a major and growing global health burden [1]. According to GLOBOCAN 2022, approximately 2.3 million new cases and 670,000 deaths were recorded globally [2]. Despite advances in screening, early detection, and treatment [3, 4], the burden of breast cancer continues to increase, driven by population aging, demographic expansion, and shifts in reproductive and lifestyle‐related risk factors [5]. These trends are particularly evident in low‐ and middle‐income countries (LMICs), where rapid epidemiological transitions occur alongside persistent disparities in healthcare access and cancer control capacity [4, 6].

Recent analyses based on GLOBOCAN 2022 have substantially advanced understanding of global breast cancer epidemiology. Kim et al. characterized international patterns and trends in breast cancer incidence and mortality across 185 countries [2], while Liao evaluated global inequalities and projected breast cancer burden through 2050 [7]. However, these studies primarily assessed breast cancer as an aggregated disease outcome and therefore did not capture potential heterogeneity between premenopausal and postmenopausal populations. Given differences in hormonal environment, risk factor profiles, tumor biology, and healthcare needs between these groups, menopausal‐status‐specific analyses are essential for identifying distinct epidemiological patterns and informing tailored cancer control strategies [8].

Menopausal status is a key yet often underexplored dimension in breast cancer epidemiology [8]. Premenopausal and postmenopausal breast cancers differ substantially in hormonal milieu, tumor biology, and risk factor profiles, and exhibit distinct epidemiological patterns [9–11]. Notably, countries with very high Human Development Index (HDI) report the highest age‐standardized incidence rates (ASIRs), whereas low‐ and medium‐HDI countries experience disproportionately high mortality [2]. These contrasting patterns reflect both differences in underlying risk factor distribution and inequities in early detection, diagnosis, and access to effective treatment [7]. Stratification by menopausal status is therefore essential for accurately characterizing the global burden and identifying targeted public health priorities [12].

The etiological determinants of premenopausal and postmenopausal breast cancer further underscore their distinct epidemiological characteristics. In premenopausal women, key risk factors include genetic predisposition (e.g., BRCA mutations), early menarche, nulliparity, delayed childbearing, and exogenous hormone use [13–15]. In contrast, postmenopausal breast cancer is more strongly associated with modifiable factors such as excess adiposity, alcohol consumption, and menopausal hormone therapy [11]. These differing risk profiles are compounded by ongoing global transitions in lifestyle and reproductive behaviors. Rapid urbanization and economic development have led to increasing prevalence of overweight and obesity worldwide, with LMICs now accounting for a substantial proportion of this burden [16]. Concurrently, declining fertility rates, delayed childbirth, and reduced breastfeeding duration are expected to further increase breast cancer risk, particularly in postmenopausal populations [17, 18].

Although a previous study based on GLOBOCAN 2018 data has provided important global estimates of breast cancer burden by menopausal status, updated and more comprehensive analyses are required [8]. Improvements in cancer registry coverage, methodological refinements in GLOBOCAN 2022, and ongoing changes in global risk factor distributions necessitate a reassessment of the current and future burden [1]. In this study, we use GLOBOCAN 2022 data to quantify the global, regional, and national burden of premenopausal and postmenopausal breast cancer. We estimate incidence, mortality, cumulative risk, and the mortality‐to‐incidence ratio (MIR), and assess their variation across countries stratified by HDI. We further analyze temporal trends and project the future burden through 2050.

## 2. Methods

### 2.1. Data Source

Breast cancer incidence and mortality data (ICD‐10 code C50) were obtained from the GLOBOCAN 2022 database, produced by the International Agency for Research on Cancer (IARC) of the World Health Organization (WHO) [1]. GLOBOCAN 2022 provides estimates for 36 cancer types across 185 countries and territories for the reference year 2022. National estimates are derived using a standardized methodology that integrates data from population‐based cancer registries, national mortality registries, and modeled approximations when high‐quality data are absent or incomplete.

Data were accessed through the Cancer Today platform (https://gco.iarc.who.int/today), while future burden projections were obtained via the Cancer Tomorrow tool (https://gco.iarc.who.int/tomorrow). Temporal trends in cancer‐specific incidence and mortality were examined using the Cancer Over Time database (https://gco.iarc.who.int/overtime), which relies on the Cancer Incidence in Five Continents (CI5 plus) database and WHO mortality statistics.

### 2.2. Definition of Menopausal Status

As population‐based registries lack individual‐level hormonal or clinical menopausal data, menopausal status was classified using age at diagnosis as a validated epidemiological proxy, consistent with prior global burden studies [8]. Women diagnosed before age 50 were classified as premenopausal, while those aged 50 and older were classified as postmenopausal. This threshold was selected based on the global average age of natural menopause, typically ranging from 45 to 55 years, with 50 years representing the mean across diverse populations [19].

To evaluate the robustness of the age‐based classification of menopausal status across populations with different ages at natural menopause, we conducted a sensitivity analysis using alternative age thresholds. Specifically, women aged 45–49 years were reclassified as postmenopausal in countries with low or medium HDI, where menopause generally occurs at younger ages, while the original cutoff of 50 years was retained for high‐HDI countries. Conversely, women aged 50–54 years were reclassified as premenopausal in very high‐HDI countries to account for the relatively later age at natural menopause reported in these populations.

### 2.3. Outcome Measures

ASIR and age‐standardized mortality rate (ASMR) were calculated per 100,000 women‐years using the Segi–Doll World Standard Population [20]. Rates were truncated to the age ranges of 15–49 years (premenopausal) and ≥ 50 years (postmenopausal).

Cumulative risk was estimated as the probability that an individual woman would develop or die from breast cancer over a defined age interval, in the hypothetical absence of competing causes of death. For premenopausal breast cancer, cumulative risk was calculated over the age range 15–49 years; for postmenopausal breast cancer, it was calculated over the age range older than 50 years. Cumulative risk was derived from age‐specific incidence and mortality rates using the standard actuarial formula [21]:

(1) Cumulative risk%=1−exp−∑iri×wi×100,

where *r*_*i*_ represents the age‐specific rate in the *i* ^th^ five‐year age group and *w*_*i*_ denotes the width of that age interval. Results are expressed as a percentage and interpreted as the cumulative lifetime probability of diagnosis or death within the specified age window.

MIR was calculated as ASMR divided by ASIR for each country, region, and HDI stratum. MIR values range from 0 to 1, with higher values indicating a greater proportion of diagnosed cases resulting in death, serving as an ecological proxy of survival and health system performance [22].

### 2.4. Developmental Stratification

HDI, developed by the United Nations, is a composite measure of a country’s social and economic development, incorporating four indicators: mean years of schooling, expected years of schooling, life expectancy at birth, and gross national income per capita. Following the approach used by the GLOBOCAN 2020 project, countries and regions were categorized into four HDI levels—low, medium, high, and very high—based on 2022 HDI estimates from the United Nations Development Programme (Supporting Table S1).

### 2.5. Projections to 2050

Future numbers of breast cancer cases and deaths were projected using the Cancer Tomorrow tool, which applies current age‐ and sex‐specific rates from GLOBOCAN 2022 to United Nations World Population Prospects demographic projections. This fixed‐rate approach assumes stable incidence and mortality rates, attributing changes solely to shifts in population size and age structure. Projections were reported separately for premenopausal (15–49 years) and postmenopausal (≥ 50 years) groups, stratified by HDI category and geographic region.

### 2.6. Trend Analysis

Temporal trends in ASIR and ASMR were characterized using the estimated annual percentage change (EAPC), calculated by fitting a log‐linear regression model of the natural logarithm of age‐standardized rates against calendar year. Trends were classified as increasing, decreasing, or stable based on whether the 95% confidence interval of the EAPC excluded zero.

### 2.7. Correlation Analysis

Spearman’s rank correlation coefficients (*ρ*) were calculated between continuous HDI scores and each outcome measure (ASIR, ASMR, MIR) to assess the association between national development and breast cancer burden. All tests were two‐tailed, with *p* < 0.05 considered statistically significant.

### 2.8. Software and Ethical Statement

All data extraction, computation, and statistical analyses were performed using R version 4.2.3 (R Foundation for Statistical Computing, Vienna, Austria). As this study is based exclusively on publicly available, aggregated, and de‐identified population‐level data from the GLOBOCAN 2022 database, no individual patient data were accessed and ethical approval was not required.

## 3. Results

### 3.1. Global Burden of Premenopausal and Postmenopausal Breast Cancer in 2022

In 2022, an estimated 2,296,607 new breast cancer cases were diagnosed worldwide, with 668,417 (29.1%) occurring in premenopausal and 1,628,190 (70.9%) in postmenopausal women (Table 1, Figure 1). The global ASIR were 32.7 and 153.9 per 100,000 for premenopausal and postmenopausal groups, corresponding to cumulative risks of 1.3% and 3.8%, respectively. Breast cancer caused 666,061 deaths globally, including 141,338 (21.2%) premenopausal and 524,723 (78.8%) postmenopausal deaths, with ASMR of 6.9 and 46.3 per 100,000 (Table 1, Figure 2). The global MIR was 21.1% for premenopausal and 30.1% for postmenopausal breast cancer, indicating higher case fatality among older women (Table 1).

**TABLE 1:** Estimated number of cases, ASIR, ASMR, cumulative risk, and mortality‐to‐incidence ratio for premenopausal and postmenopausal breast cancer in 2022.

	Incidence						Mortality						Mortality‐to‐incidence ratios	
	Premenopausal (age < 50 years)			Postmenopausal (age ≥ 50 years)			Premenopausal (age < 50 years)			Postmenopausal (age ≥ 50 years)			Premenopausal (age < 50 years)	Postmenopausal (age ≥ 50 years)
	Cases (% of total cases)	ASIR per 100,000	Cumulative risk	Cases (% of total cases)	ASIR per 100,000	Cumulative risk	Cases (% of total cases)	ASMR per 100,000	Cumulative risk	Cases (% of total cases)	ASMR per 100,000	Cumulative risk		
World	668,417 (29.1)	32.7	1.3	1,628,190 (70.9)	153.9	3.8	141,338 (21.22)	6.9	0.28	524,723 (78.78)	46.3	1.1	21.1	30.08
Africa	93,318 (47.01)	31.8	1.3	105,188 (52.99)	124.6	3.1	37,237 (40.81)	12.8	0.51	54,009 (59.19)	64.5	1.6	40.25	51.77
Eastern Africa	23,114 (48.89)	24.4	0.96	24,165 (51.11)	99.8	2.5	11,225 (44.32)	12.1	0.48	14,103 (55.68)	58.4	1.5	49.59	58.52
Middle Africa	8551 (50.77)	24.0	0.95	8292 (49.23)	91.0	2.3	4145 (45.34)	11.8	0.47	4997 (54.66)	55.2	1.4	49.17	60.66
Northern Africa	31,201 (48.02)	48.4	1.9	33,769 (51.98)	147.3	3.6	8345 (35.99)	12.9	0.52	14,840 (64.01)	63.3	1.5	26.65	42.97
Southern Africa	5208 (32.93)	27.9	1.1	10,607 (67.07)	162.5	4.0	1477 (25.95)	7.9	0.32	4214 (74.05)	63.6	1.3	28.32	39.14
Western Africa	25,244 (47.1)	31.0	1.2	28,355 (52.9)	132.1	3.3	12,045 (43.17)	15.0	0.60	15,855 (56.83)	74.6	1.9	48.39	56.47
Northern America	54,628 (17.83)	57.3	2.3	251,675 (82.17)	335.1	8.2	4802 (9.65)	5.1	0.20	44,942 (90.35)	49.3	1.1	8.9	14.71
Latin America and the Caribbean	69,076 (31.38)	36.9	1.5	151,037 (68.62)	169.5	4.2	12,681 (21.18)	6.8	0.27	47,193 (78.82)	49.4	1.2	18.43	29.14
Caribbean	4004 (26.94)	35.1	1.4	10,857 (73.06)	161.7	4.0	1136 (19.09)	10.0	0.40	4816 (80.91)	66.4	1.5	28.49	41.06
Central America	14,691 (36.88)	28.9	1.2	25,144 (63.12)	120.6	3.0	2695 (25.26)	5.3	0.22	7975 (74.74)	37.2	0.90	18.34	30.85
Southern America	50,381 (30.46)	40.4	1.6	115,036 (69.54)	186.9	4.6	8850 (20.46)	7.1	0.29	34,402 (79.54)	51.7	1.2	17.57	27.66
Asia	339,964 (34.49)	27.1	1.1	645,690 (65.51)	105.2	2.6	73,699 (23.38)	5.9	0.24	241,576 (76.62)	37.8	0.92	21.77	35.93
Eastern Asia	143,345 (29.87)	31.4	1.3	336,629 (70.13)	110.6	2.7	15,534 (15.95)	3.4	0.14	81,852 (84.05)	24.3	0.57	10.83	21.97
Southeastern Asia	62,741 (37.24)	32.9	1.3	105,750 (62.76)	128.0	3.2	15,559 (25.41)	8.2	0.33	45,662 (74.59)	54.2	1.3	24.92	42.34
South‐central Asia	107,464 (39.24)	19.9	0.79	166,372 (60.76)	84.6	2.1	35,975 (26.58)	6.8	0.27	99,350 (73.42)	50.4	1.3	34.17	59.57
Western Asia	26,414 (41.69)	35.6	1.4	36,939 (58.31)	139.9	3.5	6631 (31.07)	9.0	0.36	14,712 (68.93)	53.6	1.3	25.28	38.31
Europe	105,186 (18.87)	50.7	2.0	452,338 (81.13)	253.7	6.2	12,032 (8.33)	5.7	0.23	132,407 (91.67)	58.8	1.4	11.24	23.18
Eastern Europe	34,674 (21.21)	39.4	1.6	128,799 (78.79)	194.9	5.0	5275 (10.56)	5.9	0.24	44,698 (89.44)	57.8	1.4	14.97	29.66
Northern Europe	15,289 (17.12)	54.8	2.2	74,034 (82.88)	319.7	7.8	1501 (8.06)	5.4	0.22	17,128 (91.94)	55.4	1.2	9.85	17.33
Southern Europe	25,704 (20.63)	60.9	2.4	98,915 (79.37)	259.6	6.4	2393 (7.61)	5.4	0.22	29,057 (92.39)	55.8	1.3	8.87	21.49
Western Europe	29,519 (16.39)	60.3	2.4	150,590 (83.61)	301.4	7.3	2863 (6.45)	5.8	0.23	41,524 (93.55)	62.7	1.4	9.62	20.8
Oceania	6245 (21.91)	55.5	2.2	22,262 (78.09)	321.4	7.8	887 (16.18)	7.9	0.32	4596 (83.82)	57.8	1.3	14.23	17.98
Australia and New Zealand	5095 (19.76)	61.4	2.5	20,691 (80.24)	351.0	8.5	429 (10.33)	5.2	0.21	3723 (89.67)	51.4	1.2	8.47	14.64
Melanesia	1028 (44.81)	38.1	1.5	1266 (55.19)	152.8	3.8	430 (36.47)	16.0	0.64	749 (63.53)	94.7	2.2	41.99	61.98
Micronesia	34 (25.56)	26.6	1.1	99 (74.44)	154.2	3.5	6 (11.32)	4.9	0.20	47 (88.68)	71.7	1.5	18.42	46.5
Polynesia	88 (29.93)	52.1	2.1	206 (70.07)	256.7	6.1	22 (22.22)	13.1	0.53	77 (77.78)	94.4	2.1	25.14	36.77
Human development index (HDI)														
Very high HDI countries	224,946 (20.59)	50.7	2.0	867,695 (79.41)	254.0	6.3	25,652 (10.47)	5.7	0.23	219,390 (89.53)	52.0	1.2	11.24	20.47
High HDI countries	249,628 (33.83)	32.0	1.3	488,234 (66.17)	115.5	2.9	43,333 (22.18)	5.5	0.22	151,998 (77.82)	34.0	0.81	17.19	29.44
Medium HDI countries	127,951 (38.65)	21.9	0.87	203,067 (61.35)	94.9	2.4	42,922 (27.91)	7.4	0.30	110,891 (72.09)	51.6	1.3	33.79	54.37
Low HDI countries	65,622 (48.94)	27.1	1.1	68,457 (51.06)	104.0	2.6	29,337 (41.06)	12.4	0.49	42,118 (58.94)	64.6	1.6	45.76	62.12

Abbreviations: ASIR, age‐standardized incidence rate; ASMR, age‐standardized mortality rate.

**FIGURE 1:**
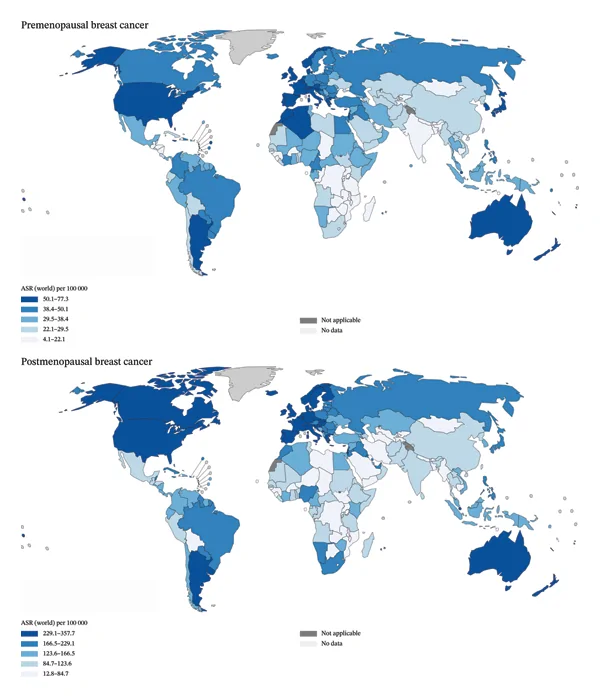
Global distribution of age‐standardized incidence rates (ASIR) of breast cancer in 2022 among premenopausal and postmenopausal women. Data were obtained from the GLOBOCAN 2022 database, compiled by the International Agency for Research on Cancer (IARC) and accessed through the global cancer observatory.

**FIGURE 2:**
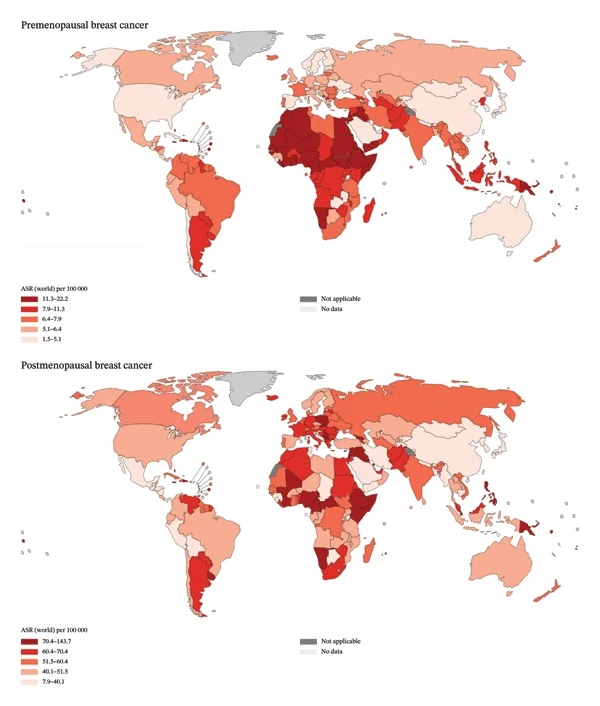
Global distribution of age‐standardized mortality rates (ASMR) for breast cancer among premenopausal and postmenopausal women in 2022. Estimates are derived from the GLOBOCAN 2022 database, compiled by the International Agency for Research on Cancer (IARC) and accessed through the Global Cancer Observatory.

Country‐level data further revealed substantial heterogeneity in age distribution (Supporting Table S2). While postmenopausal breast cancer predominated globally, premenopausal cases accounted for over half of all cases in several LMICs, such as Afghanistan (54.8%), Bangladesh (54.0%), and Ethiopia (56.4%). In contrast, high‐income countries (HICs) such as Canada, Australia, and Western Europe reported markedly lower proportions of premenopausal disease (generally < 20%). Across all countries, ASIRs and cumulative risks were consistently higher in postmenopausal women, particularly in HICs where postmenopausal cumulative risk exceeded 5%–8%.

### 3.2. Regional Distribution of Incidence

Substantial regional heterogeneity was observed (Table 1). Premenopausal cases accounted for 47.0% of incident breast cancer in Africa but only 17.8% in Northern America and 18.9% in Europe. Elevated premenopausal proportions were also observed in Western Asia (41.7%), Southeastern Asia (39.2%), and South‐central Asia (37.2%). Premenopausal ASIRs were highest in Northern America (57.3 per 100,000), Oceania (55.5), and Europe (50.7), and lowest in Asia (27.1). Postmenopausal ASIRs exhibited a steeper gradient: Northern America (335.1), Oceania (321.4), and Europe (253.7) had the highest rates, whereas Asia had the lowest (105.2). Subregionally, Australia and New Zealand exhibited the highest postmenopausal ASIR globally (351.0 per 100,000), consistent with country‐level observations (Supporting Table S2).

### 3.3. Regional Distribution of Mortality and MIRs

Mortality patterns diverged from incidence (Table 1). Africa recorded the highest ASMRs for premenopausal (12.8) and postmenopausal (64.5) disease, whereas Northern America had the lowest premenopausal ASMR (5.1) and among the lowest postmenopausal ASMRs (49.3). Country‐level analyses further underscored pronounced disparities (Supporting Table S2). Despite lower incidence, many LMICs exhibited disproportionately high mortality burdens, particularly among premenopausal women, with several African countries reporting > 40% of deaths occurring before age 50.

MIRs reflected disparities in healthcare access: Africa had the highest MIRs (pre: 40.3%, post: 51.8%), Northern America had the lowest premenopausal MIR (8.9%), and Australia/New Zealand had the lowest postmenopausal MIR (14.6%). At the country level, MIRs were exceptionally high in resource‐limited settings (e.g., > 50% in parts of sub‐Saharan Africa), but remained low in HICs (generally < 20%), reflecting substantial differences in early detection and treatment access.

### 3.4. Distribution by HDI

Breast cancer burden varied markedly with HDI (Table 1). Very high HDI countries exhibited the highest ASIRs (pre: 50.7, post: 254.0) but the lowest MIRs (pre: 11.2%, post: 20.5%), reflecting widespread screening and treatment availability. Low HDI countries had lower ASIRs (pre: 27.1, post: 104.0) but substantially higher MIRs (pre: 45.8%, post: 62.1%), highlighting poor survival outcomes. The proportion of premenopausal cases increased with decreasing HDI, from 20.6% in very high HDI countries to 48.9% in low HDI countries.

### 3.5. Correlation With HDI

Spearman’s rank correlation analyses demonstrated significant associations between HDI and breast cancer burden metrics across 185 countries (Figure 3). Cumulative incidence risk positively correlated with HDI (premenopausal *ρ* = 0.671, postmenopausal *ρ* = 0.773; *p* < 0.0001), whereas premenopausal cumulative mortality was inversely associated with HDI (*ρ* = −0.506, *p* < 0.0001). Postmenopausal mortality showed no significant correlation (*ρ* = 0.058, *p* = 0.447). MIR exhibited the strongest inverse associations with HDI (premenopausal *ρ* = −0.924, postmenopausal *ρ* = −0.845; *p* < 0.0001), confirming that higher national development is associated with lower case fatality.

**FIGURE 3:**
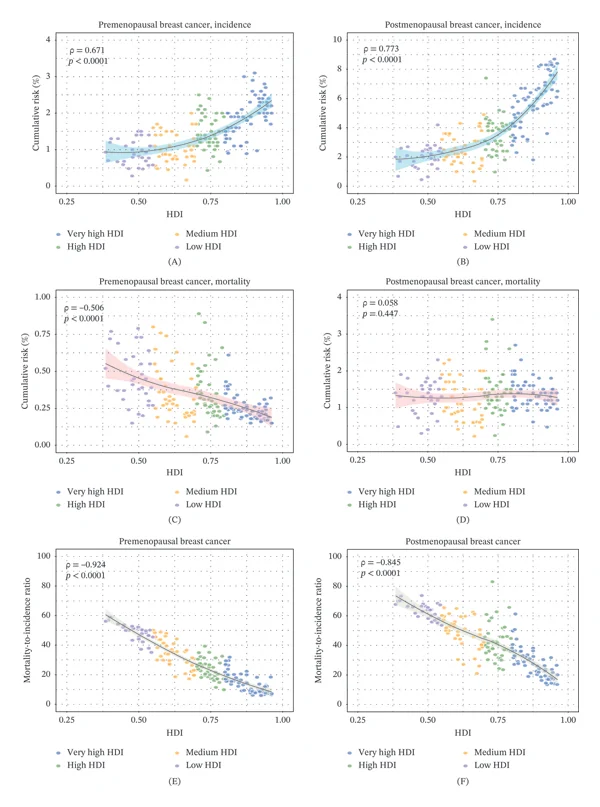
Associations between breast cancer burden metrics and the human development index (HDI) by menopausal status. (A) Cumulative incidence risk versus HDI in premenopausal women. (B) Cumulative incidence risk versus HDI in postmenopausal women. (C) Cumulative mortality risk versus HDI in premenopausal women. (D) Cumulative mortality risk versus HDI in postmenopausal women. (E) Mortality‐to‐incidence ratio (MIR) versus HDI in premenopausal women. (F) MIR versus HDI in postmenopausal women. MIR = mortality‐to‐incidence ratio.

### 3.6. Temporal Trends in Incidence and Mortality

During 2003–2017, premenopausal incidence increased most rapidly in the Republic of Korea, Japan, Latvia, Turkey, and Lithuania, and declined in Qatar, the Philippines, Colombia, and Argentina (Figure 4A). Postmenopausal incidence rose sharply in Qatar, the Republic of Korea, Japan, Thailand, India, and China, whereas declines occurred in Argentina and the Philippines (Figure 4B).

**FIGURE 4:**
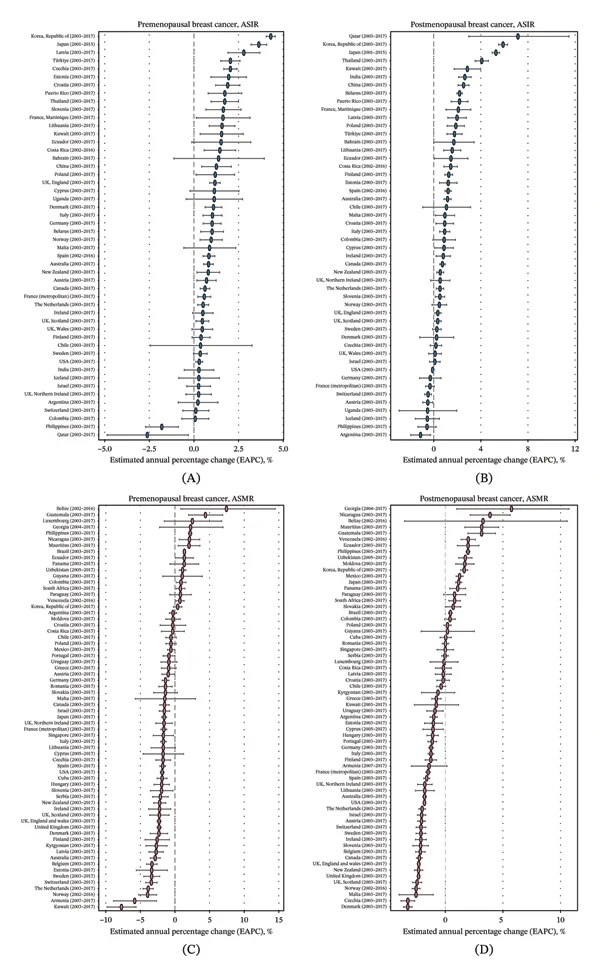
Estimated annual percentage change (EAPC) in breast cancer incidence and mortality during 2003–2017, stratified by menopausal status. (A) EAPC in age‐standardized incidence rates (ASIR) of premenopausal breast cancer. (B) EAPC in age‐standardized incidence rates (ASIR) of postmenopausal breast cancer. (C) EAPC in age‐standardized mortality rates (ASMR) of premenopausal breast cancer. (D) EAPC in age‐standardized mortality rates (ASMR) of postmenopausal breast cancer.

Premenopausal mortality declined in most populations, particularly in Kuwait and Armenia, but increased in Belize, Guatemala, Georgia, the Philippines, and Nicaragua (Figure 4C). Postmenopausal mortality declined in Denmark, Malta, Switzerland, Norway, and the United Kingdom, but increased in Georgia, Nicaragua, Guatemala, Venezuela, Ecuador, and the Philippines (Figure 4D), reflecting widening survival disparities.

### 3.7. Projections to 2050

Under fixed‐rate projections, global premenopausal cases are expected to reach 795,908 (+19.1%) and postmenopausal cases 2,756,889 (+69.3%), with deaths increasing to 168,669 (+19.3%) and 969,442 (+84.8%), respectively (Table 2). Africa is projected to experience the largest relative increases (pre: +106.5%, post: +153.2%), whereas Europe is expected to see declines in premenopausal incidence (−21.1%) and mortality (−21.4%). Asia shows a modest rise in premenopausal cases (+7.6%) but a substantial increase in postmenopausal cases (+65.8%), primarily in South‐central and Western Asia. Latin America, Northern America, and Oceania are projected to experience moderate‐to‐high increases, with Melanesia showing particularly steep growth. Overall, these projections indicate that the future global burden of breast cancer will be increasingly driven by postmenopausal disease, while LMICs will continue to face a disproportionate burden of early‐onset disease and poor survival outcomes (Supporting Table S3).

**TABLE 2:** Projected number of premenopausal and postmenopausal breast cancer cases in 2050 and percentage change relative to 2022.

	Incidence				Mortality			
	Premenopausal (age < 50 years)		Postmenopausal (age ≥ 50 years)		Premenopausal (age < 50 years)		Postmenopausal (age ≥ 50 years)	
	Cases (% of total cases)	Percentage change in number of cases (%)	Cases (% of total cases)	Percentage change in number of cases (%)	Cases (% of total cases)	Percentage change in number of cases (%)	Cases (% of total cases)	Percentage change in number of cases (%)
World	795,908 (22.4)	19.07	2,756,889 (77.6)	69.32	168,669 (242.23)	19.34	969,442 (85.18)	84.75
Africa	192,735 (32.29)	106.54	404,114 (67.71)	284.18	77,088 (35.58)	107.02	139,573 (64.42)	158.43
Eastern Africa	52,984 (43.09)	129.23	69,968 (56.91)	189.54	25,971 (38.85)	131.37	40,887 (61.15)	189.92
Middle Africa	21,887 (48.85)	155.96	22,920 (51.15)	176.41	10,626 (43.46)	156.36	13,825 (56.54)	176.67
Northern Africa	45,828 (38.8)	46.88	72,300 (61.2)	114.1	12,269 (25.82)	47.02	35,253 (74.18)	137.55
Southern Africa	6339 (22.26)	21.72	22,139 (77.74)	108.72	1797 (15.41)	21.67	9861 (84.59)	134.01
Western Africa	56,507 (43.97)	123.84	72,008 (56.03)	153.95	27,047 (40.06)	124.55	40,473 (59.94)	155.27
Northern America	62,658 (15.9)	14.7	331,302 (84.1)	31.64	5557 (7.3)	15.72	70,534 (92.7)	56.94
Latin America and the Caribbean	72,807 (14.74)	5.4	421,234 (85.26)	178.89	13,656 (12.69)	7.69	93,998 (87.31)	99.18
Caribbean	4283 (20.21)	6.97	16,906 (79.79)	55.72	1235 (13.12)	8.71	8181 (86.88)	69.87
Central America	16,769 (24.7)	14.14	51,112 (75.3)	103.28	3173 (15.59)	17.74	17,178 (84.41)	115.4
Southern America	51,253 (19.9)	1.73	206,293 (80.1)	79.33	9176 (12.02)	3.68	67,141 (87.98)	95.17
Asia	365,823 (18.38)	7.61	1,624,588 (81.62)	151.6	79,780 (15.12)	8.25	447,961 (84.88)	85.43
Eastern Asia	107,621 (20.12)	−24.92	427,354 (79.88)	26.95	11,644 (8.21)	−25.04	130,123 (91.79)	58.97
Southeastern Asia	67,899 (26.98)	8.22	183,752 (73.02)	73.76	16,968 (16.01)	9.06	89,042 (83.99)	95
South‐central Asia	139,112 (27.93)	29.45	358,996 (72.07)	115.78	47,602 (17.33)	32.32	227,052 (82.67)	128.54
Western Asia	36,303 (30.23)	37.44	83,797 (69.77)	126.85	9165 (20.13)	38.21	36,358 (79.87)	147.13
Europe	82,986 (9.62)	−21.11	779,869 (90.38)	72.41	9455 (5.21)	−21.42	172,056 (94.79)	29.94
Eastern Europe	23,979 (14.86)	−30.84	137,429 (85.14)	6.7	3609 (6.44)	−31.58	52,403 (93.56)	17.24
Northern Europe	15,234 (14.28)	−0.36	91,481 (85.72)	23.57	1500 (5.79)	−0.07	24,405 (94.21)	42.49
Southern Europe	18,225 (14.06)	−29.1	111,414 (85.94)	12.64	1679 (4.06)	−29.84	39,710 (95.94)	36.66
Western Europe	27,140 (13.35)	−8.06	176,109 (86.65)	16.95	2640 (4.49)	−7.79	56,128 (95.51)	35.17
Oceania	8164 (12.41)	30.73	57,610 (87.59)	158.78	1160 (11.78)	30.78	8687 (88.22)	89.01
Australia and New Zealand	5877 (15.35)	15.35	32,420 (84.65)	56.69	497 (6.86)	15.85	6752 (93.14)	81.36
Melanesia	1832 (36.15)	78.21	3236 (63.85)	155.61	768 (27.18)	78.6	2058 (72.82)	174.77
Micronesia	44 (19.64)	29.41	180 (80.36)	81.82	8 (7.08)	33.33	105 (92.92)	123.4
Polynesia	109 (20.57)	23.86	421 (79.43)	104.37	27 (13.43)	22.73	174 (86.57)	125.97
Human development index (HDI)								
Very high HDI countries	205,232 (15.66)	−8.76	1,105,208 (84.34)	27.37	23,441 (6.82)	−8.62	320,381 (93.18)	46.03
High HDI countries	224,471 (23.54)	−10.08	728,919 (76.46)	49.3	39,090 (12.64)	−9.79	270,213 (87.36)	77.77
Medium HDI countries	172,030 (28.23)	34.45	437,458 (71.77)	115.43	58,502 (18.78)	36.3	253,028 (81.22)	128.18
Low HDI countries	147,939 (44.95)	125.44	181,163 (55.05)	164.64	66,749 (37.37)	127.53	111,856 (62.63)	165.58

### 3.8. Sensitivity Analysis

The sensitivity analysis using alternative region‐specific age thresholds yielded findings that were highly consistent with those of the primary analysis (Supporting Figures S1–S3). ASIRs remained positively associated with HDI in both premenopausal and postmenopausal women, with a stronger association observed among postmenopausal women (Supporting Figure S1A–B). ASMRs showed only weak associations with HDI, with no significant relationship observed for premenopausal breast cancer and a modest positive association for postmenopausal disease (Supporting Figure S1C–D). Similar patterns were observed for cumulative risk (Supporting Figure S2). Cumulative incidence risk increased progressively with HDI in both menopausal groups, whereas cumulative mortality risk generally declined with increasing HDI. In addition, MIR remained strongly inversely associated with HDI for both premenopausal and postmenopausal breast cancer (Supporting Figure S3).

## 4. Discussion

Using the most recent estimates from the IARC GLOBOCAN 2022 database, this study provides a comprehensive and stratified assessment of the global burden of breast cancer by menopausal status. By jointly examining incidence, mortality, cumulative risk, and MIR, our analysis offers a more nuanced characterization of global breast cancer epidemiology than studies treating breast cancer as a single entity. Overall, our findings confirm persistent and substantial global disparities in breast cancer outcomes, while highlighting shifting geographic patterns and a rapidly increasing future burden.

Mark Heer and colleagues have documented international variation in incidence by menopausal status, but has largely focused on incidence alone [8]. By integrating mortality metrics, survival proxies, temporal trends, and projections, our study extends this evidence base and provides a more comprehensive assessment of the evolving global landscape. Notably, we identify emerging geographic shifts, with several Asian countries experiencing some of the fastest increases in breast cancer incidence. This pattern supports the concept of a global “cancer transition”, whereby countries undergoing socioeconomic development increasingly adopt risk profiles and disease patterns historically observed in high‐income settings [23, 24].

The global burden of breast cancer continues to rise in both premenopausal and postmenopausal women. Although relative increases in recent years appear modest, the absolute growth in cases and deaths is substantial and is projected to accelerate. This trend is consistent with findings from the Global Burden of Disease Study, which attribute rising cancer burden primarily to population growth and aging [25, 26]. The more pronounced increase among postmenopausal women likely reflects both demographic aging and cumulative exposure to established risk factors, including obesity, physical inactivity, delayed childbearing, and reduced parity [27, 28]. At the same time, the persistent and, in some regions, increasing burden of premenopausal breast cancer is of particular concern. Early‐onset disease is often associated with more aggressive tumor subtypes and greater socioeconomic consequences [29]. While the underlying drivers remain incompletely understood, changes in reproductive patterns, increasing obesity at younger ages, and improved detection may all contribute [30–32]. These findings highlight the need for further integrative research combining epidemiological, behavioral, and molecular perspectives.

There was stark disparity between incidence and mortality across levels of development. Countries with higher HDI exhibit substantially higher incidence rates but markedly lower MIRs, whereas low‐HDI countries experience lower incidence yet disproportionately high mortality. This divergence underscores the critical role of health system capacity in shaping cancer outcomes [33]. Differences in stage at diagnosis, access to timely and multimodal treatment, availability of radiotherapy, and overall health system infrastructure likely account for much of this inequality [34–36]. The strong inverse association between HDI and MIR further reinforces that survival disparities are closely linked to broader socioeconomic development. Importantly, these inequities are evident in both premenopausal and postmenopausal populations, indicating systemic gaps in access to care across the age spectrum.

The rapid increase in incidence observed in several Asian countries further illustrates the impact of epidemiological transition. Urbanization, declining fertility, delayed age at first birth, and increasing prevalence of obesity and sedentary lifestyles are likely contributing to these trends [37–39]. These findings suggest that countries currently undergoing rapid socioeconomic change may face a substantial escalation in breast cancer burden in the coming decades [40]. Anticipating this shift is essential for health system planning, including investment in diagnostic capacity, oncology workforce development, and treatment infrastructure.

Encouragingly, mortality rates continue to decline in many HICs, reflecting the combined benefits of organized screening programs and advances in treatment, including endocrine therapy, targeted therapies, and improved chemotherapy regimens [26]. However, increasing mortality trends in some middle‐income settings indicate that these gains have not been equitably distributed. In resource‐limited settings, rising incidence may translate directly into increased mortality due to delayed diagnosis and inadequate treatment access [41, 42]. This divergence highlights the ongoing need to strengthen cancer control systems globally [43].

These findings align with broader global health frameworks emphasizing equity in cancer control. The Women, Power, and Cancer: a Lancet Commission underscores how gender inequities, structural barriers, and unequal access to health services continue to shape cancer outcomes worldwide [44]. Our results reinforce this perspective by demonstrating persistent survival gaps between high‐ and low‐resource settings. Addressing these disparities will require coordinated, system‐level interventions, including expanding access to early diagnosis, ensuring availability of essential cancer medicines and technologies, and strengthening health system governance [45–47].

From a policy perspective, our findings highlight the importance of context‐specific breast cancer control strategies. In high‐income settings, where incidence is high and health systems are well developed, population‐based mammographic screening has contributed to mortality reduction [48]. In contrast, in many LMICs, resource constraints may necessitate prioritizing early diagnosis, awareness campaigns, and timely referral pathways over population‐wide screening [49–52]. Additionally, the observed increase in premenopausal breast cancer in certain regions raises important questions regarding the appropriateness of current screening age thresholds and supports consideration of risk‐stratified approaches [53–55]. However, such strategies must be carefully evaluated in relation to cost‐effectiveness and health system capacity.

Projection analyses indicate that the global breast cancer burden will increase substantially by 2050, with the largest relative increases expected in regions undergoing rapid demographic and epidemiological transitions, particularly in Africa. These projected increases are driven primarily by population growth and aging, but may be further amplified by changes in risk factor profiles [56]. These findings underscore the urgency of strengthening cancer control systems in regions where infrastructure remains limited. Strategic investments in cancer registries, diagnostic services, treatment facilities, and workforce capacity will be essential to mitigate the future burden.

Our findings complement previous GLOBOCAN 2022–based assessments by demonstrating that the global breast cancer burden is heterogeneous across menopausal groups. Specifically, premenopausal breast cancer accounts for a substantially greater proportion of the overall breast cancer burden in many low‐ and middle‐income regions, particularly in Africa and parts of Asia, despite their lower overall incidence rates. This pattern identifies a population subgroup with potentially distinct prevention, early detection, and treatment needs. Furthermore, although higher‐HDI countries generally exhibit higher incidence and lower MIRs, the magnitude and geographic distribution of these disparities differ between premenopausal and postmenopausal breast cancer, suggesting that differences in healthcare access, diagnostic capacity, and treatment availability may affect outcomes differently across menopausal groups. Menopausal status–specific projections further demonstrate that the future global increase in breast cancer burden will be driven predominantly by postmenopausal disease in absolute numbers as a consequence of population aging, while several rapidly transitioning regions are expected to experience disproportionately large increases in premenopausal breast cancer. These findings provide more actionable evidence for cancer control planning than aggregate projections alone. Overall, stratification by menopausal status reveals distinct geographic patterns, socioeconomic gradients, and future burden trajectories that may be obscured when breast cancer is considered as a single disease entity.

Several limitations should be considered. First, the classification of menopausal status using an age threshold of 50 years represents an important limitation. Although this approach is widely used in global cancer epidemiological studies and is supported by the overall international distribution of menopausal age, it cannot fully capture population‐specific differences in reproductive aging. Earlier menopause in some low‐ and middle‐income populations may lead to potential overestimation of premenopausal breast cancer burden among women aged 45–49 years. To evaluate the potential impact of this assumption, we performed a sensitivity analysis using alternative region‐specific age thresholds that better reflect population differences in the timing of natural menopause. The findings were highly consistent with those of the primary analysis, demonstrating that our results were robust to alternative definitions of menopausal status. Nevertheless, age‐based classification cannot fully capture biological menopausal status, and future studies incorporating individual‐level reproductive information are warranted to further refining estimates. Second, GLOBOCAN estimates rely on modeled data in settings lacking high‐quality cancer registries, potentially affecting accuracy in some regions. Third, projections assume stable age‐specific rates and do not account for future changes in risk factors, screening, or treatment. Finally, although MIR is a useful ecological proxy for survival, it may be influenced by lead‐time bias and should not be interpreted as a direct measure of survival.

## 5. Conclusions

In conclusion, this study provides updated global evidence on the burden of breast cancer by menopausal status and highlights persistent and substantial inequalities in outcomes across countries and development levels. While incidence remains highest in highly developed regions, survival gaps continue to disadvantage low‐resource settings. The projected increase in global burden—particularly in rapidly transitioning regions—emphasizes the need for strengthened, equitable cancer control strategies.

## Author Contributions

Rongbo Ding: conceptualization, methodology, data curation, formal analysis, visualization, writing–original draft, and writing–review and editing. Hongyan Jia: conceptualization, methodology, data curation, validation, supervision, writing–original draft, and writing–review and editing.

## Funding

This study was supported by the Wu Jieping Medical Foundation (No. 320.6750.2025‐21‐119).

## Ethics Statement

This study was based exclusively on publicly available, de‐identified data obtained from the GLOBOCAN 2022 database of the International Agency for Research on Cancer. As no individual‐level identifiable information was used, ethical approval and informed consent were not required in accordance with institutional and international guidelines.

## Consent

Please see the Ethics Statement.

## Conflicts of Interest

The authors declare no conflicts of interest.

## Supporting Information

Additional supporting information can be found online in the Supporting Information section.

## Supporting information


**Supporting Information 1** Supporting Figure S1. Associations between breast cancer age‐standardized rates and the Human Development Index (HDI) by menopausal status. (A) Age‐standardized incidence rate (ASIR) versus HDI in premenopausal women. (B) ASIR versus HDI in postmenopausal women. (C) Age‐standardized mortality rate (ASMR) versus HDI in premenopausal women. (D) ASMR versus HDI in postmenopausal women. Supporting Figure S2. Associations between breast cancer cumulative risk and the Human Development Index (HDI) by menopausal status. (A) Cumulative incidence risk versus HDI in premenopausal women. (B) Cumulative incidence risk versus HDI in postmenopausal women. (C) Cumulative mortality risk versus HDI in premenopausal women. (D) Cumulative mortality risk versus HDI in postmenopausal women. Supporting Figure S3. Associations between the breast cancer mortality‐to‐incidence ratio (MIR) and the Human Development Index (HDI) by menopausal status. (A) MIR versus HDI in premenopausal women. (B) MIR versus HDI in postmenopausal women.

**Supporting Information 2** Supporting Table S1. Human Development Index (HDI) quintiles for countries and territories estimated by the United Nations Development Programme. Supporting Table S2. Estimated number of cases, ASIR, ASMR, cumulative risk, and mortality‐to‐incidence ratio for premenopausal and postmenopausal breast cancer in 2022, by country and territory. Supporting Table S3. Projected number of cases, ASIR, ASMR, cumulative risk, and mortality‐to‐incidence ratio for premenopausal and postmenopausal breast cancer in 2050, by country and territory.

**Supporting Information 3** STROBE Checklist.

## Data Availability

The data and materials presented in the study are included in the article/Supporting Information. All data used in this study will also be made available upon request to the corresponding author.
