# Supplementary material for: Burden and Projections of Premenopausal and Postmenopausal Breast Cancer Worldwide: A Comprehensive Analysis of GLOBOCAN 2022 Data
Source: Breast J. 2026 Nov 4;2026:1290471. doi: 10.1155/tbj/1290471 (PMC13736169; doi:10.1155/tbj/1290471)
Supplement: Supplementary file 2 [file TBJ-2026-1290471-s001.docx]

**Supplementary Figure S1**. Associations between breast cancer age-standardized rates and the Human Development Index (HDI) by menopausal status. (A) Age-standardized incidence rate (ASIR) versus HDI in premenopausal women. (B) ASIR versus HDI in postmenopausal women. (C) Age-standardized mortality rate (ASMR) versus HDI in premenopausal women. (D) ASMR versus HDI in postmenopausal women.

**Supplementary Figure S2**. Associations between breast cancer cumulative risk and the Human Development Index (HDI) by menopausal status. (A) Cumulative incidence risk versus HDI in premenopausal women. (B) Cumulative incidence risk versus HDI in postmenopausal women. (C) Cumulative mortality risk versus HDI in premenopausal women. (D) Cumulative mortality risk versus HDI in postmenopausal women.

**Supplementary Figure S3**. Associations between the breast cancer mortality-to-incidence ratio (MIR) and the Human Development Index (HDI) by menopausal status. (A) MIR versus HDI in premenopausal women. (B) MIR versus HDI in postmenopausal women.
