# Supplementary material for: Burden and Projections of Premenopausal and Postmenopausal Breast Cancer Worldwide: A Comprehensive Analysis of GLOBOCAN 2022 Data
Source: Breast J. 2026 Nov 4;2026:1290471. doi: 10.1155/tbj/1290471 (PMC13736169; doi:10.1155/tbj/1290471)
Supplement: Supplementary file 3 [file TBJ-2026-1290471-s002.docx]

Supplementary Table S1. Human Development Index (HDI) quintiles for countries and territories estimated by the United Nations Development Programme

| **HDI category** | **Locations included based on HDI values estimates from the United Nations Development Programme.** |
| --- | --- |
| Very high HDI countries | Argentina, Australia, Austria, Bahamas, Bahrain, Barbados, Belgium, Brunei Darussalam, Belarus, Canada, Chile, Costa Rica, Croatia, Cyprus, Czechia, Denmark, Estonia, Finland, France (metropolitan), Georgia, Germany, Greece, Hungary, Iceland, Ireland, Israel, Italy, Japan, Kazakhstan, Korea, Republic of, Kuwait, Latvia, Lithuania, Luxembourg, Malaysia, Malta, Montenegro, Oman, The Netherlands, New Zealand, Norway, Panama, Poland, Portugal, Qatar, Romania, Russian Federation, Saudi Arabia, Serbia, Singapore, Slovakia, Slovenia, Spain, Sweden, Switzerland, Thailand, Trinidad and Tobago, United Arab Emirates, Türkiye, United Kingdom, United States of America, Uruguay |
| High HDI countries | Albania, Algeria, Azerbaijan, Armenia, Bosnia Herzegovina, Botswana, Brazil, Belize, Bulgaria, Sri Lanka, China, Colombia, Cuba, Dominican Republic, Ecuador, Fiji, Gaza Strip and West Bank, Guyana, Indonesia, Iran, Islamic Republic of, Jamaica, Jordan, Kyrgyzstan, Lebanon, Libya, Maldives, Mauritius, Mexico, Mongolia, Republic of Moldova, Paraguay, Peru, Philippines, Saint Lucia, Viet Nam, South Africa, Tunisia, Turkmenistan, Ukraine, North Macedonia, Egypt, Uzbekistan, Samoa |
| Medium HDI countries | Angola, Bangladesh, Bhutan, Bolivia (Plurinational State of), Solomon Islands, Myanmar, Cambodia, Cameroon, Cape Verde, Comoros, Congo, Republic of, El Salvador, Equatorial Guinea, Gabon, Ghana, Guatemala, Haiti, Honduras, India, Iraq, Kenya, Lao People's Democratic Republic, Morocco, Namibia, Nepal, Vanuatu, Nicaragua, Papua New Guinea, Timor-Leste, Sao Tome and Principe, Zimbabwe, Suriname, Eswatini, Syrian Arab Republic, Tajikistan, Uganda, Venezuela, Zambia |
| Low HDI countries | Afghanistan, Burundi, Central African Republic, Chad, Congo, Democratic Republic of, Benin, Ethiopia, Eritrea, Djibouti, The Republic of the Gambia, Guinea, Côte d'Ivoire, Lesotho, Liberia, Madagascar, Malawi, Mali, Mauritania, Mozambique, Niger, Nigeria, Pakistan, Guinea-Bissau, Rwanda, Senegal, Sierra Leone, Somalia, South Sudan, Sudan, Togo, Tanzania, United Republic of, Burkina Faso, Yemen |

**Supplementary Table S2.** Estimated number of cases, ASIR, ASMR, cumulative risk, and mortality-to-incidence ratio for premenopausal and postmenopausal breast cancer in 2022, by country and territory

|  | Incidence | | | | | |  | Mortality | | | | | |  | Mortality-to-incidence ratios | |
| --- | --- | --- | --- | --- | --- | --- | --- | --- | --- | --- | --- | --- | --- | --- | --- | --- |
|  | Premenopausal (age <50 years) | | | Postmenopausal (age ≥50 years) | | |  | Premenopausal (age <50 years) | | | Postmenopausal (age ≥50 years) | | |  | Premenopausal (age <50 years) | Postmenopausal (age ≥50 years) |
|  | Cases (% of total cases) | ASIR per 100,000 | Cumulative risk | Cases (% of total cases) | ASIR per 100,000 | Cumulative risk |  | Cases (% of total cases) | ASMR per 100,000 | Cumulative risk | Cases (% of total cases) | ASMR per 100,000 | Cumulative risk |  |  |  |
| Afghanistan | 1942 (54.78) | 25.4 | 1.0 | 1603 (45.22) | 84.9 | 2.1 |  | 796 (40.61) | 11.0 | 0.44 | 1164 (59.39) | 62.6 | 1.6 |  | 43.31 | 73.73 |
| Albania | 330 (32.32) | 48.7 | 1.9 | 691 (67.68) | 135.9 | 3.4 |  | 32 (7.86) | 4.8 | 0.20 | 375 (92.14) | 60.7 | 1.5 |  | 9.86 | 44.67 |
| Algeria | 7605 (52.11) | 60.4 | 2.4 | 6990 (47.89) | 161.4 | 4.0 |  | 1917 (39.19) | 15.2 | 0.61 | 2975 (60.81) | 66.7 | 1.6 |  | 25.17 | 41.33 |
| Angola | 1510 (52.1) | 22.2 | 0.87 | 1388 (47.9) | 92.7 | 2.4 |  | 603 (45.24) | 9.2 | 0.36 | 730 (54.76) | 48.9 | 1.3 |  | 41.44 | 52.75 |
| Argentina | 6291 (29.08) | 50.7 | 2.0 | 15340 (70.92) | 232.5 | 6.0 |  | 1064 (16.53) | 8.5 | 0.34 | 5372 (83.47) | 67.0 | 1.6 |  | 16.77 | 28.82 |
| Armenia | 179 (17.67) | 20.4 | 0.81 | 834 (82.33) | 148.2 | 3.8 |  | 44 (9.71) | 5.1 | 0.20 | 409 (90.29) | 68.0 | 1.7 |  | 25 | 45.88 |
| Australia | 4301 (19.61) | 61.1 | 2.4 | 17630 (80.39) | 357.7 | 8.7 |  | 333 (9.81) | 4.7 | 0.19 | 3060 (90.19) | 49.9 | 1.1 |  | 7.69 | 13.95 |
| Austria | 1196 (19.99) | 50.5 | 2.0 | 4787 (80.01) | 223.9 | 5.6 |  | 122 (6.82) | 5.1 | 0.21 | 1667 (93.18) | 58.8 | 1.4 |  | 10.1 | 26.26 |
| Azerbaijan | 855 (38.95) | 28.5 | 1.1 | 1340 (61.05) | 94.8 | 2.4 |  | 228 (28.72) | 7.6 | 0.31 | 566 (71.28) | 40.4 | 1.0 |  | 26.67 | 42.62 |
| Bahamas | 60 (34.88) | 51.0 | 2.0 | 112 (65.12) | 198.2 | 4.7 |  | 18 (20.69) | 15.1 | 0.61 | 69 (79.31) | 121.5 | 2.7 |  | 29.61 | 61.3 |
| Bahrain | 115 (35.49) | 29.6 | 1.2 | 209 (64.51) | 219.9 | 5.4 |  | 22 (22.92) | 5.9 | 0.24 | 74 (77.08) | 83.8 | 1.9 |  | 19.93 | 38.11 |
| Bangladesh | 7015 (54.03) | 14.6 | 0.58 | 5969 (45.97) | 40.2 | 0.99 |  | 2467 (40.04) | 5.2 | 0.21 | 3695 (59.96) | 25.1 | 0.61 |  | 35.62 | 62.44 |
| Barbados | 38 (26.21) | 50.1 | 2.0 | 107 (73.79) | 166.0 | 3.9 |  | 13 (14.77) | 16.3 | 0.66 | 75 (85.23) | 109.1 | 2.6 |  | 32.53 | 65.72 |
| Belarus | 1052 (20.3) | 38.0 | 1.5 | 4131 (79.7) | 189.7 | 4.9 |  | 171 (12.36) | 6.1 | 0.25 | 1213 (87.64) | 51.5 | 1.3 |  | 16.05 | 27.15 |
| Belgium | 2073 (18.42) | 69.3 | 2.8 | 9182 (81.58) | 352.2 | 8.5 |  | 136 (5.85) | 4.5 | 0.18 | 2188 (94.15) | 60.0 | 1.3 |  | 6.49 | 17.04 |
| Belize | 27 (31.76) | 25.1 | 0.99 | 58 (68.24) | 172.2 | 4.3 |  | 6 (28.57) | 5.6 | 0.22 | 15 (71.43) | 44.7 | 1.1 |  | 22.31 | 25.96 |
| Benin | 562 (42.29) | 23.1 | 0.93 | 767 (57.71) | 109.8 | 2.8 |  | 270 (36.78) | 11.2 | 0.45 | 464 (63.22) | 67.0 | 1.7 |  | 48.48 | 61.02 |
| Bhutan | 8 (50) | 4.1 | 0.17 | 8 (50) | 12.9 | 0.34 |  | 3 (37.5) | 1.5 | 0.06 | 5 (62.5) | 7.9 | 0.22 |  | 36.59 | 61.24 |
| Bolivia (Plurinational State of) | 881 (52.47) | 29.3 | 1.1 | 798 (47.53) | 62.1 | 1.4 |  | 167 (32.49) | 5.7 | 0.23 | 347 (67.51) | 23.7 | 0.46 |  | 19.45 | 38.16 |
| Bosnia Herzegovina | 342 (22.22) | 38.6 | 1.5 | 1197 (77.78) | 164.9 | 4.2 |  | 51 (9.04) | 5.7 | 0.23 | 513 (90.96) | 61.1 | 1.5 |  | 14.77 | 37.05 |
| Botswana | 114 (44.36) | 16.9 | 0.68 | 143 (55.64) | 73.6 | 1.8 |  | 35 (38.46) | 5.2 | 0.21 | 56 (61.54) | 28.6 | 0.68 |  | 30.77 | 38.86 |
| Brazil | 27289 (28.81) | 42.4 | 1.7 | 67437 (71.19) | 211.8 | 5.2 |  | 4736 (21.34) | 7.3 | 0.30 | 17453 (78.66) | 51.5 | 1.2 |  | 17.22 | 24.32 |
| Brunei Darussalam | 51 (38.93) | 37.1 | 1.5 | 80 (61.07) | 160.0 | 4.0 |  | 8 (22.86) | 5.8 | 0.24 | 27 (77.14) | 57.1 | 1.4 |  | 15.63 | 35.69 |
| Bulgaria | 740 (20.8) | 38.5 | 1.5 | 2818 (79.2) | 169.7 | 4.3 |  | 132 (9.44) | 6.6 | 0.27 | 1267 (90.56) | 61.0 | 1.4 |  | 17.14 | 35.95 |
| Burkina Faso | 685 (49.93) | 17.1 | 0.68 | 687 (50.07) | 65.1 | 1.7 |  | 365 (44.62) | 9.2 | 0.37 | 453 (55.38) | 43.3 | 1.2 |  | 53.8 | 66.51 |
| Burundi | 395 (45.93) | 16.9 | 0.67 | 465 (54.07) | 85.1 | 2.1 |  | 206 (40.31) | 9.2 | 0.37 | 305 (59.69) | 56.0 | 1.4 |  | 54.44 | 65.8 |
| Cambodia | 967 (45.79) | 22.1 | 0.89 | 1145 (54.21) | 71.3 | 1.7 |  | 328 (35.77) | 7.6 | 0.31 | 589 (64.23) | 37.4 | 0.83 |  | 34.39 | 52.45 |
| Cameroon | 2222 (52.82) | 39.3 | 1.6 | 1985 (47.18) | 147.0 | 3.6 |  | 1079 (47.22) | 19.2 | 0.76 | 1206 (52.78) | 90.3 | 2.3 |  | 48.85 | 61.43 |
| Canada | 5098 (16.02) | 49.7 | 2.0 | 26725 (83.98) | 321.3 | 7.9 |  | 551 (8.07) | 5.4 | 0.22 | 6276 (91.93) | 53.8 | 1.0 |  | 10.87 | 16.74 |
| Cape Verde | 35 (52.24) | 23.7 | 0.96 | 32 (47.76) | 59.0 | 1.6 |  | 7 (30.43) | 4.9 | 0.19 | 16 (69.57) | 27.0 | 0.59 |  | 20.68 | 45.76 |
| Central African Republic | 298 (51.92) | 33.3 | 1.3 | 276 (48.08) | 107.0 | 2.6 |  | 175 (46.54) | 19.6 | 0.77 | 201 (53.46) | 78.5 | 1.9 |  | 58.86 | 73.36 |
| Chad | 535 (72.1) | 17.6 | 0.70 | 207 (27.9) | 27.9 | 0.68 |  | 302 (67.71) | 10.0 | 0.40 | 144 (32.29) | 19.5 | 0.48 |  | 56.82 | 69.89 |
| Chile | 1488 (26.38) | 27.4 | 1.1 | 4152 (73.62) | 124.0 | 3.1 |  | 247 (13.92) | 4.5 | 0.18 | 1528 (86.08) | 40.3 | 0.92 |  | 16.42 | 32.5 |
| China | 111983 (31.36) | 28.0 | 1.1 | 245134 (68.64) | 96.6 | 2.4 |  | 12824 (17.1) | 3.2 | 0.13 | 62159 (82.9) | 22.7 | 0.54 |  | 11.43 | 23.5 |
| Colombia | 5662 (33.28) | 38.7 | 1.5 | 11352 (66.72) | 158.6 | 3.9 |  | 977 (20.56) | 6.7 | 0.27 | 3775 (79.44) | 50.0 | 1.2 |  | 17.31 | 31.53 |
| Comoros | 28 (43.08) | 14.2 | 0.56 | 37 (56.92) | 72.6 | 1.8 |  | 12 (35.29) | 6.4 | 0.25 | 22 (64.71) | 43.3 | 0.90 |  | 45.07 | 59.64 |
| Congo, Democratic Republic of | 3439 (46.61) | 20.0 | 0.79 | 3940 (53.39) | 83.3 | 2.1 |  | 1768 (41.56) | 10.4 | 0.41 | 2486 (58.44) | 52.7 | 1.3 |  | 52 | 63.27 |
| Congo, Republic of | 244 (46.04) | 20.2 | 0.81 | 286 (53.96) | 84.9 | 2.1 |  | 107 (44.4) | 8.8 | 0.36 | 134 (55.6) | 40.9 | 1.0 |  | 43.56 | 48.17 |
| Costa Rica | 339 (25.22) | 23.2 | 0.94 | 1005 (74.78) | 130.8 | 3.3 |  | 66 (15.6) | 4.5 | 0.18 | 357 (84.4) | 42.0 | 0.97 |  | 19.4 | 32.11 |
| Côte d'Ivoire | 2206 (57.06) | 40.3 | 1.6 | 1660 (42.94) | 128.4 | 3.2 |  | 1103 (52.72) | 20.2 | 0.80 | 989 (47.28) | 77.3 | 1.9 |  | 50.12 | 60.2 |
| Croatia | 557 (17.92) | 51.7 | 2.1 | 2551 (82.08) | 246.5 | 6.1 |  | 50 (8.17) | 4.7 | 0.19 | 562 (91.83) | 46.7 | 1.2 |  | 9.09 | 18.95 |
| Cuba | 745 (14.45) | 25.5 | 1.0 | 4412 (85.55) | 173.8 | 4.3 |  | 143 (8.04) | 4.8 | 0.20 | 1636 (91.96) | 57.5 | 1.3 |  | 18.82 | 33.08 |
| Cyprus | 268 (26.27) | 73.8 | 2.9 | 752 (73.73) | 342.9 | 8.2 |  | 16 (6.72) | 4.5 | 0.18 | 222 (93.28) | 82.1 | 1.8 |  | 6.1 | 23.94 |
| Czechia | 1561 (20.19) | 48.4 | 1.9 | 6171 (79.81) | 244.0 | 6.0 |  | 125 (7.31) | 3.7 | 0.15 | 1584 (92.69) | 49.3 | 1.1 |  | 7.64 | 20.2 |
| Denmark | 806 (15.33) | 56.2 | 2.2 | 4453 (84.67) | 339.6 | 8.2 |  | 59 (5.3) | 4.1 | 0.17 | 1055 (94.7) | 60.4 | 1.4 |  | 7.3 | 17.79 |
| Djibouti | 99 (55) | 36.4 | 1.4 | 81 (45) | 101.2 | 2.5 |  | 50 (49.02) | 18.5 | 0.73 | 52 (50.98) | 65.3 | 1.6 |  | 50.82 | 64.53 |
| Dominican Republic | 1337 (41.23) | 46.2 | 1.8 | 1906 (58.77) | 153.5 | 3.8 |  | 414 (28.43) | 14.5 | 0.58 | 1042 (71.57) | 79.5 | 1.9 |  | 31.39 | 51.79 |
| Ecuador | 1371 (35.13) | 29.0 | 1.2 | 2532 (64.87) | 126.4 | 3.1 |  | 252 (21.84) | 5.4 | 0.22 | 902 (78.16) | 42.6 | 1.0 |  | 18.62 | 33.7 |
| Egypt | 12781 (47.61) | 49.1 | 1.9 | 14064 (52.39) | 156.6 | 3.8 |  | 3485 (36.32) | 13.4 | 0.54 | 6111 (63.68) | 66.7 | 1.5 |  | 27.29 | 42.59 |
| El Salvador | 517 (32.07) | 28.4 | 1.1 | 1095 (67.93) | 128.8 | 3.1 |  | 88 (26.51) | 4.9 | 0.20 | 244 (73.49) | 27.0 | 0.61 |  | 17.25 | 20.96 |
| Equatorial Guinea | 110 (63.22) | 39.2 | 1.5 | 64 (36.78) | 108.9 | 2.6 |  | 52 (59.77) | 18.8 | 0.74 | 35 (40.23) | 60.9 | 1.5 |  | 47.96 | 55.92 |
| Eritrea | 277 (55.18) | 36.1 | 1.4 | 225 (44.82) | 100.4 | 2.5 |  | 140 (48.11) | 18.5 | 0.73 | 151 (51.89) | 66.7 | 1.7 |  | 51.25 | 66.43 |
| Estonia | 151 (17.62) | 41.7 | 1.7 | 706 (82.38) | 213.3 | 5.3 |  | 16 (5.99) | 4.3 | 0.18 | 251 (94.01) | 55.5 | 1.3 |  | 10.31 | 26.02 |
| Eswatini | 48 (45.28) | 17.2 | 0.68 | 58 (54.72) | 88.8 | 2.2 |  | 19 (40.43) | 6.8 | 0.28 | 28 (59.57) | 39.6 | 0.86 |  | 39.53 | 44.59 |
| Ethiopia | 9534 (56.4) | 38.3 | 1.5 | 7370 (43.6) | 110.3 | 2.8 |  | 4908 (50.99) | 20.0 | 0.79 | 4718 (49.01) | 70.7 | 1.8 |  | 52.22 | 64.1 |
| Fiji | 123 (42.56) | 52.4 | 2.1 | 166 (57.44) | 172.9 | 4.3 |  | 48 (26.37) | 20.6 | 0.83 | 134 (73.63) | 143.7 | 3.4 |  | 39.31 | 83.11 |
| Finland | 646 (12.01) | 48.0 | 1.9 | 4732 (87.99) | 344.0 | 8.4 |  | 56 (6.37) | 4.2 | 0.17 | 823 (93.63) | 49.3 | 1.2 |  | 8.75 | 14.33 |
| France (metropolitan) | 12708 (19.35) | 77.3 | 3.1 | 52950 (80.65) | 337.6 | 8.3 |  | 1087 (7.37) | 6.5 | 0.26 | 13652 (92.63) | 63.0 | 1.4 |  | 8.41 | 18.66 |
| France, Guadeloupe | 55 (23.31) | 57.1 | 2.3 | 181 (76.69) | 192.3 | 4.8 |  | 11 (15.07) | 11.2 | 0.46 | 62 (84.93) | 56.0 | 1.3 |  | 19.61 | 29.12 |
| France, La Réunion | 106 (27.75) | 44.9 | 1.8 | 276 (72.25) | 169.6 | 4.2 |  | 22 (24.44) | 9.2 | 0.37 | 68 (75.56) | 38.0 | 0.87 |  | 20.49 | 22.41 |
| France, Martinique | 56 (19.44) | 63.4 | 2.5 | 232 (80.56) | 230.6 | 5.7 |  | 10 (13.51) | 11.2 | 0.46 | 64 (86.49) | 53.9 | 1.3 |  | 17.67 | 23.37 |
| French Guyana | 29 (33.72) | 35.9 | 1.4 | 57 (66.28) | 190.5 | 4.3 |  | 6 (25) | 7.4 | 0.30 | 18 (75) | 61.5 | 1.2 |  | 20.61 | 32.28 |
| French Polynesia | 42 (31.11) | 51.4 | 2.1 | 93 (68.89) | 233.5 | 5.4 |  | 10 (22.22) | 12.2 | 0.50 | 35 (77.78) | 86.2 | 1.9 |  | 23.74 | 36.92 |
| Gabon | 184 (56.97) | 33.7 | 1.3 | 139 (43.03) | 99.5 | 2.5 |  | 56 (49.56) | 10.3 | 0.41 | 57 (50.44) | 41.0 | 0.98 |  | 30.56 | 41.21 |
| Gaza Strip and West Bank | 373 (45.05) | 33.2 | 1.3 | 455 (54.95) | 150.4 | 3.8 |  | 117 (35.14) | 10.7 | 0.43 | 216 (64.86) | 72.4 | 1.8 |  | 32.23 | 48.14 |
| Georgia | 356 (20.58) | 32.9 | 1.3 | 1374 (79.42) | 164.7 | 4.1 |  | 95 (11.03) | 8.7 | 0.35 | 766 (88.97) | 82.0 | 2.0 |  | 26.44 | 49.79 |
| Germany | 9361 (12.65) | 45.8 | 1.8 | 64652 (87.35) | 272.8 | 6.6 |  | 1201 (5.83) | 5.9 | 0.24 | 19400 (94.17) | 64.7 | 1.5 |  | 12.88 | 23.72 |
| Ghana | 2599 (51.71) | 36.0 | 1.4 | 2427 (48.29) | 112.8 | 2.8 |  | 1145 (48.33) | 15.9 | 0.63 | 1224 (51.67) | 57.8 | 1.5 |  | 44.17 | 51.24 |
| Greece | 1631 (18.15) | 57.6 | 2.3 | 7356 (81.85) | 270.8 | 6.6 |  | 156 (6.42) | 5.3 | 0.21 | 2275 (93.58) | 60.8 | 1.3 |  | 9.2 | 22.45 |
| Guam | 11 (20.75) | 26.8 | 1.1 | 42 (79.25) | 155.1 | 3.6 |  | 2 (8.33) | 5.1 | 0.21 | 22 (91.67) | 72.5 | 1.6 |  | 19.03 | 46.74 |
| Guatemala | 923 (41.13) | 20.4 | 0.82 | 1321 (58.87) | 91.7 | 2.2 |  | 166 (31.2) | 3.8 | 0.15 | 366 (68.8) | 24.9 | 0.59 |  | 18.63 | 27.15 |
| Guinea | 327 (45.67) | 12.2 | 0.48 | 389 (54.33) | 49.9 | 1.2 |  | 158 (40.72) | 6.1 | 0.24 | 230 (59.28) | 29.7 | 0.72 |  | 50 | 59.52 |
| Guinea-Bissau | 97 (52.15) | 22.0 | 0.87 | 89 (47.85) | 82.1 | 2.0 |  | 49 (44.95) | 11.3 | 0.45 | 60 (55.05) | 55.8 | 1.3 |  | 51.36 | 67.97 |
| Guyana | 65 (28.51) | 34.3 | 1.4 | 163 (71.49) | 177.7 | 4.3 |  | 17 (25.37) | 9.0 | 0.36 | 50 (74.63) | 53.8 | 1.3 |  | 26.24 | 30.28 |
| Haiti | 588 (43.39) | 19.5 | 0.77 | 767 (56.61) | 78.0 | 1.8 |  | 215 (28.51) | 7.4 | 0.30 | 539 (71.49) | 53.4 | 1.2 |  | 37.95 | 68.46 |
| Honduras | 490 (40.53) | 19.3 | 0.77 | 719 (59.47) | 89.2 | 2.3 |  | 199 (39.48) | 7.9 | 0.31 | 305 (60.52) | 36.7 | 0.92 |  | 40.93 | 41.14 |
| Hungary | 1345 (17.48) | 46.8 | 1.9 | 6349 (82.52) | 267.3 | 6.7 |  | 169 (7.55) | 5.7 | 0.23 | 2068 (92.45) | 70.4 | 1.7 |  | 12.18 | 26.34 |
| Iceland | 38 (18.54) | 42.4 | 1.7 | 167 (81.46) | 251.5 | 6.1 |  | 7 (10.77) | 7.8 | 0.32 | 58 (89.23) | 66.6 | 1.4 |  | 18.4 | 26.48 |
| India | 68306 (35.58) | 18.5 | 0.74 | 123667 (64.42) | 87.6 | 2.2 |  | 24073 (24.49) | 6.6 | 0.27 | 74243 (75.51) | 52.3 | 1.3 |  | 35.68 | 59.7 |
| Indonesia | 27375 (41.32) | 34.6 | 1.4 | 38878 (58.68) | 124.1 | 3.1 |  | 6878 (30.45) | 8.7 | 0.35 | 15713 (69.55) | 50.5 | 1.3 |  | 25.14 | 40.69 |
| Iran, Islamic Republic of | 8616 (55.64) | 31.4 | 1.2 | 6868 (44.36) | 75.3 | 1.8 |  | 1784 (33.64) | 6.4 | 0.26 | 3519 (66.36) | 39.4 | 0.92 |  | 20.38 | 52.32 |
| Iraq | 4080 (47.3) | 43.6 | 1.7 | 4546 (52.7) | 177.7 | 4.3 |  | 1273 (37.75) | 13.7 | 0.55 | 2099 (62.25) | 84.0 | 2.0 |  | 31.42 | 47.27 |
| Ireland | 868 (23.54) | 56.2 | 2.3 | 2820 (76.46) | 319.5 | 7.6 |  | 108 (12.23) | 6.9 | 0.28 | 775 (87.77) | 69.1 | 1.5 |  | 12.28 | 21.63 |
| Israel | 1108 (23.99) | 50.5 | 2.0 | 3510 (76.01) | 270.1 | 6.5 |  | 151 (12.51) | 6.9 | 0.28 | 1056 (87.49) | 64.2 | 1.4 |  | 13.66 | 23.77 |
| Italy | 10299 (17.92) | 64.5 | 2.6 | 47181 (82.08) | 277.1 | 6.7 |  | 939 (6.08) | 5.6 | 0.23 | 14516 (93.92) | 60.5 | 1.3 |  | 8.68 | 21.83 |
| Jamaica | 524 (39.49) | 63.6 | 2.5 | 803 (60.51) | 199.9 | 4.7 |  | 181 (26.46) | 22.2 | 0.89 | 503 (73.54) | 121.8 | 2.8 |  | 34.91 | 60.93 |
| Japan | 19476 (21.19) | 59.5 | 2.4 | 72440 (78.81) | 226.1 | 5.6 |  | 1420 (8.05) | 4.1 | 0.17 | 16218 (91.95) | 38.3 | 0.92 |  | 6.89 | 16.94 |
| Jordan | 1259 (48.46) | 49.4 | 2.0 | 1339 (51.54) | 179.0 | 4.5 |  | 316 (39.4) | 12.5 | 0.50 | 486 (60.6) | 66.1 | 1.7 |  | 25.3 | 36.93 |
| Kazakhstan | 1157 (25.32) | 22.3 | 0.90 | 3413 (74.68) | 130.1 | 3.3 |  | 303 (19.25) | 5.9 | 0.24 | 1271 (80.75) | 47.2 | 1.2 |  | 26.46 | 36.28 |
| Kenya | 3147 (43.45) | 25.6 | 1.0 | 4096 (56.55) | 141.1 | 3.5 |  | 1360 (40.02) | 11.2 | 0.45 | 2038 (59.98) | 70.5 | 1.7 |  | 43.75 | 49.96 |
| Korea, Democratic People's Republic of | 2077 (34.95) | 29.1 | 1.2 | 3866 (65.05) | 89.9 | 2.3 |  | 666 (37.08) | 9.4 | 0.37 | 1130 (62.92) | 26.3 | 0.67 |  | 32.3 | 29.25 |
| Korea, Republic of | 9705 (39.18) | 66.3 | 2.6 | 15066 (60.82) | 144.9 | 3.6 |  | 602 (20.67) | 3.9 | 0.16 | 2311 (79.33) | 19.4 | 0.47 |  | 5.88 | 13.39 |
| Kuwait | 352 (39.46) | 26.8 | 1.1 | 540 (60.54) | 183.5 | 4.4 |  | 74 (29.02) | 5.4 | 0.22 | 181 (70.98) | 71.8 | 1.5 |  | 20.15 | 39.13 |
| Kyrgyzstan | 343 (40.26) | 20.3 | 0.81 | 509 (59.74) | 77.0 | 1.9 |  | 64 (23.7) | 4.0 | 0.16 | 206 (76.3) | 31.3 | 0.79 |  | 19.7 | 40.65 |
| Lao People's Democratic Republic | 520 (50.34) | 27.4 | 1.1 | 513 (49.66) | 90.9 | 2.2 |  | 135 (35.16) | 7.3 | 0.30 | 249 (64.84) | 44.8 | 1.1 |  | 26.64 | 49.28 |
| Latvia | 236 (18.69) | 47.1 | 1.9 | 1027 (81.31) | 214.3 | 5.5 |  | 32 (7.44) | 6.2 | 0.25 | 398 (92.56) | 66.2 | 1.6 |  | 13.16 | 30.89 |
| Lebanon | 808 (37.41) | 43.1 | 1.7 | 1352 (62.59) | 183.7 | 4.6 |  | 217 (27.78) | 11.6 | 0.47 | 564 (72.22) | 73.5 | 1.8 |  | 26.91 | 40.01 |
| Lesotho | 59 (31.55) | 11.6 | 0.47 | 128 (68.45) | 66.1 | 1.5 |  | 24 (25) | 4.9 | 0.20 | 72 (75) | 36.9 | 0.84 |  | 42.24 | 55.82 |
| Liberia | 325 (52.25) | 29.2 | 1.2 | 297 (47.75) | 96.3 | 2.4 |  | 165 (47.01) | 15.0 | 0.60 | 186 (52.99) | 60.6 | 1.5 |  | 51.37 | 62.93 |
| Libya | 648 (55.43) | 29.3 | 1.2 | 521 (44.57) | 83.0 | 2.1 |  | 174 (37.83) | 7.9 | 0.32 | 286 (62.17) | 47.0 | 1.1 |  | 26.96 | 56.63 |
| Lithuania | 298 (17.34) | 44.4 | 1.8 | 1421 (82.66) | 205.0 | 5.2 |  | 45 (8.74) | 6.6 | 0.27 | 470 (91.26) | 56.2 | 1.4 |  | 14.86 | 27.41 |
| Luxembourg | 115 (20.35) | 60.7 | 2.4 | 450 (79.65) | 349.8 | 8.3 |  | 10 (9.52) | 5.2 | 0.21 | 95 (90.48) | 62.7 | 1.4 |  | 8.57 | 17.92 |
| Madagascar | 1259 (43.79) | 20.8 | 0.82 | 1616 (56.21) | 100.7 | 2.5 |  | 563 (37.58) | 9.5 | 0.38 | 935 (62.42) | 58.6 | 1.4 |  | 45.67 | 58.19 |
| Malawi | 790 (51.2) | 19.7 | 0.78 | 753 (48.8) | 77.7 | 1.9 |  | 391 (44.84) | 9.9 | 0.39 | 481 (55.16) | 49.9 | 1.2 |  | 50.25 | 64.22 |
| Malaysia | 3316 (39.62) | 36.4 | 1.5 | 5053 (60.38) | 141.2 | 3.5 |  | 964 (27.34) | 10.7 | 0.43 | 2562 (72.66) | 70.3 | 1.6 |  | 29.4 | 49.79 |
| Maldives | 42 (49.41) | 32.4 | 1.3 | 43 (50.59) | 119.8 | 2.9 |  | 8 (30.77) | 6.5 | 0.26 | 18 (69.23) | 50.2 | 1.2 |  | 20.06 | 41.9 |
| Mali | 1248 (54.81) | 32.3 | 1.3 | 1029 (45.19) | 109.4 | 2.7 |  | 669 (49.63) | 17.5 | 0.69 | 679 (50.37) | 72.7 | 1.8 |  | 54.18 | 66.45 |
| Malta | 72 (18.65) | 59.6 | 2.4 | 314 (81.35) | 285.6 | 7.1 |  | 7 (8.64) | 5.7 | 0.23 | 74 (91.36) | 55.1 | 1.2 |  | 9.56 | 19.29 |
| Mauritania | 307 (51.17) | 28.7 | 1.1 | 293 (48.83) | 102.4 | 2.5 |  | 148 (46.98) | 13.9 | 0.55 | 167 (53.02) | 58.6 | 1.5 |  | 48.43 | 57.23 |
| Mauritius | 132 (24.58) | 36.3 | 1.4 | 405 (75.42) | 174.5 | 4.3 |  | 36 (17.22) | 9.8 | 0.40 | 173 (82.78) | 70.8 | 1.7 |  | 27 | 40.57 |
| Mexico | 11632 (37.47) | 31.2 | 1.2 | 19411 (62.53) | 123.3 | 3.1 |  | 2005 (24.47) | 5.4 | 0.22 | 6190 (75.53) | 38.3 | 0.94 |  | 17.31 | 31.06 |
| Mongolia | 104 (45.81) | 10.6 | 0.43 | 123 (54.19) | 37.3 | 0.97 |  | 22 (39.29) | 2.3 | 0.09 | 34 (60.71) | 10.3 | 0.24 |  | 21.7 | 27.61 |
| Montenegro | 81 (19.47) | 46.6 | 1.9 | 335 (80.53) | 268.5 | 6.7 |  | 14 (9.09) | 7.9 | 0.32 | 140 (90.91) | 96.8 | 2.3 |  | 16.95 | 36.05 |
| Morocco | 5314 (41.66) | 50.8 | 2.0 | 7441 (58.34) | 167.4 | 4.1 |  | 1184 (29.28) | 11.3 | 0.45 | 2860 (70.72) | 62.6 | 1.5 |  | 22.24 | 37.4 |
| Mozambique | 978 (47.75) | 15.0 | 0.59 | 1070 (52.25) | 61.8 | 1.4 |  | 506 (41.92) | 7.8 | 0.31 | 701 (58.08) | 40.7 | 0.92 |  | 52 | 65.86 |
| Myanmar | 3520 (46.05) | 22.1 | 0.87 | 4124 (53.95) | 62.8 | 1.5 |  | 1051 (32.13) | 6.6 | 0.27 | 2220 (67.87) | 34.3 | 0.82 |  | 29.86 | 54.62 |
| Namibia | 234 (42.31) | 38.0 | 1.5 | 319 (57.69) | 176.6 | 4.3 |  | 86 (37.89) | 14.1 | 0.57 | 141 (62.11) | 78.1 | 1.9 |  | 37.11 | 44.22 |
| Nepal | 1023 (45.37) | 11.5 | 0.45 | 1232 (54.63) | 43.7 | 1.0 |  | 344 (29.94) | 3.9 | 0.16 | 805 (70.06) | 28.4 | 0.62 |  | 33.91 | 64.99 |
| New Caledonia | 52 (29.55) | 63.2 | 2.5 | 124 (70.45) | 296.0 | 7.2 |  | 9 (25.71) | 10.8 | 0.44 | 26 (74.29) | 57.7 | 1.3 |  | 17.09 | 19.49 |
| New Zealand | 794 (20.6) | 63.2 | 2.5 | 3061 (79.4) | 317.1 | 7.6 |  | 96 (12.65) | 7.6 | 0.31 | 663 (87.35) | 59.0 | 1.4 |  | 12.03 | 18.61 |
| Nicaragua | 367 (30.36) | 19.8 | 0.80 | 842 (69.64) | 128.0 | 3.1 |  | 103 (28.14) | 5.6 | 0.22 | 263 (71.86) | 39.5 | 0.95 |  | 28.28 | 30.86 |
| Niger | 1363 (63.54) | 31.2 | 1.2 | 782 (36.46) | 70.9 | 1.7 |  | 801 (59.16) | 18.4 | 0.72 | 553 (40.84) | 49.8 | 1.3 |  | 58.97 | 70.24 |
| Nigeria | 13820 (42.82) | 33.9 | 1.4 | 18458 (57.18) | 174.5 | 4.2 |  | 6361 (38.95) | 15.8 | 0.64 | 9971 (61.05) | 95.4 | 2.3 |  | 46.61 | 54.67 |
| North Macedonia | 303 (27.65) | 50.1 | 2.0 | 793 (72.35) | 203.7 | 5.1 |  | 53 (13.98) | 8.6 | 0.35 | 326 (86.02) | 76.4 | 1.9 |  | 17.17 | 37.51 |
| Norway | 795 (17.75) | 55.8 | 2.2 | 3685 (82.25) | 341.1 | 8.4 |  | 52 (6.8) | 3.6 | 0.15 | 713 (93.2) | 46.1 | 0.96 |  | 6.45 | 13.52 |
| Oman | 350 (63.75) | 32.0 | 1.3 | 199 (36.25) | 91.5 | 2.3 |  | 111 (55.78) | 10.2 | 0.41 | 88 (44.22) | 40.5 | 0.98 |  | 31.88 | 44.26 |
| Pakistan | 14639 (47.72) | 28.2 | 1.1 | 16037 (52.28) | 102.1 | 2.6 |  | 4839 (31.11) | 9.9 | 0.39 | 10713 (68.89) | 68.8 | 1.8 |  | 35.11 | 67.38 |
| Panama | 396 (36.36) | 33.5 | 1.3 | 693 (63.64) | 124.8 | 3.1 |  | 62 (20.88) | 5.2 | 0.21 | 235 (79.12) | 39.5 | 0.91 |  | 15.52 | 31.65 |
| Papua New Guinea | 765 (46) | 35.5 | 1.4 | 898 (54) | 143.1 | 3.6 |  | 347 (38.56) | 16.2 | 0.65 | 553 (61.44) | 93.8 | 2.2 |  | 45.63 | 65.55 |
| Paraguay | 857 (41.4) | 46.9 | 1.8 | 1213 (58.6) | 176.5 | 4.3 |  | 147 (24.18) | 8.5 | 0.35 | 461 (75.82) | 63.5 | 1.4 |  | 18.12 | 35.98 |
| Peru | 3266 (41.89) | 34.8 | 1.4 | 4531 (58.11) | 111.2 | 2.8 |  | 524 (26.86) | 5.6 | 0.23 | 1427 (73.14) | 33.3 | 0.79 |  | 16.09 | 29.95 |
| Philippines | 11638 (35.19) | 41.9 | 1.7 | 21434 (64.81) | 199.0 | 4.9 |  | 2634 (22.21) | 9.7 | 0.39 | 9223 (77.79) | 83.7 | 2.0 |  | 23.15 | 42.06 |
| Poland | 5001 (20.48) | 44.9 | 1.8 | 19417 (79.52) | 219.7 | 5.5 |  | 730 (8.37) | 6.4 | 0.26 | 7993 (91.63) | 71.3 | 1.6 |  | 14.25 | 32.45 |
| Portugal | 2135 (23.84) | 75.2 | 3.0 | 6819 (76.16) | 259.5 | 6.4 |  | 212 (9.59) | 7.1 | 0.29 | 1999 (90.41) | 55.3 | 1.2 |  | 9.44 | 21.31 |
| Puerto Rico | 294 (15.79) | 38.4 | 1.5 | 1568 (84.21) | 217.6 | 5.5 |  | 55 (10.91) | 7.1 | 0.29 | 449 (89.09) | 54.3 | 1.3 |  | 18.49 | 24.95 |
| Qatar | 139 (56.05) | 27.3 | 1.1 | 109 (43.95) | 128.2 | 3.2 |  | 28 (45.16) | 5.6 | 0.23 | 34 (54.84) | 43.1 | 1.1 |  | 20.51 | 33.62 |
| Republic of Moldova | 411 (25.37) | 32.3 | 1.3 | 1209 (74.63) | 153.8 | 4.1 |  | 88 (13.04) | 7.0 | 0.28 | 587 (86.96) | 70.2 | 1.9 |  | 21.67 | 45.64 |
| Romania | 2555 (20.14) | 47.9 | 1.9 | 10130 (79.86) | 228.8 | 5.8 |  | 359 (9.26) | 6.5 | 0.26 | 3518 (90.74) | 66.4 | 1.6 |  | 13.57 | 29.02 |
| Russian Federation | 17462 (22.15) | 39.5 | 1.6 | 61376 (77.85) | 191.6 | 5.0 |  | 2728 (12.34) | 6.1 | 0.25 | 19387 (87.66) | 53.0 | 1.4 |  | 15.44 | 27.66 |
| Rwanda | 437 (54.69) | 14.4 | 0.57 | 362 (45.31) | 45.7 | 1.2 |  | 201 (50.12) | 6.7 | 0.27 | 200 (49.88) | 25.3 | 0.63 |  | 46.53 | 55.36 |
| Saint Lucia | 31 (46.97) | 54.8 | 2.2 | 35 (53.03) | 124.2 | 2.9 |  | 7 (30.43) | 12.4 | 0.50 | 16 (69.57) | 51.6 | 1.2 |  | 22.63 | 41.55 |
| Samoa | 21 (28) | 53.4 | 2.1 | 54 (72) | 313.0 | 7.4 |  | 5 (20.83) | 12.7 | 0.52 | 19 (79.17) | 109.2 | 2.6 |  | 23.78 | 34.89 |
| Sao Tome and Principe | 9 (56.25) | 19.2 | 0.74 | 7 (43.75) | 52.6 | 1.6 |  | 3 (42.86) | 6.8 | 0.28 | 4 (57.14) | 31.3 | 1.1 |  | 35.42 | 59.51 |
| Saudi Arabia | 2238 (59.25) | 22.4 | 0.89 | 1539 (40.75) | 71.6 | 1.8 |  | 507 (48.47) | 5.0 | 0.20 | 539 (51.53) | 26.0 | 0.61 |  | 22.32 | 36.31 |
| Senegal | 964 (52.48) | 25.3 | 0.99 | 873 (47.52) | 87.3 | 2.2 |  | 454 (46.56) | 12.1 | 0.48 | 521 (53.44) | 52.4 | 1.3 |  | 47.83 | 60.02 |
| Serbia | 885 (18.06) | 36.4 | 1.5 | 4014 (81.94) | 213.1 | 5.4 |  | 154 (8.35) | 6.2 | 0.25 | 1691 (91.65) | 77.3 | 1.9 |  | 17.03 | 36.27 |
| Sierra Leone | 124 (60.78) | 7.2 | 0.28 | 80 (39.22) | 17.6 | 0.45 |  | 65 (55.08) | 3.8 | 0.15 | 53 (44.92) | 11.7 | 0.30 |  | 52.78 | 66.48 |
| Singapore | 969 (27.15) | 53.6 | 2.1 | 2600 (72.85) | 231.8 | 5.7 |  | 117 (11.89) | 6.4 | 0.26 | 867 (88.11) | 73.5 | 1.8 |  | 11.94 | 31.71 |
| Slovakia | 680 (18.64) | 40.9 | 1.6 | 2969 (81.36) | 236.4 | 5.9 |  | 99 (7.98) | 5.9 | 0.24 | 1142 (92.02) | 76.1 | 1.7 |  | 14.43 | 32.19 |
| Slovenia | 309 (18.61) | 55.2 | 2.2 | 1351 (81.39) | 276.1 | 6.9 |  | 29 (6.17) | 5.0 | 0.20 | 441 (93.83) | 62.9 | 1.4 |  | 9.06 | 22.78 |
| Solomon Islands | 67 (51.94) | 42.4 | 1.7 | 62 (48.06) | 142.1 | 3.6 |  | 20 (39.22) | 12.7 | 0.50 | 31 (60.78) | 71.2 | 1.7 |  | 29.95 | 50.11 |
| Somalia | 887 (48.98) | 31.4 | 1.2 | 924 (51.02) | 116.2 | 2.9 |  | 506 (43.43) | 18.4 | 0.73 | 659 (56.57) | 83.2 | 2.1 |  | 58.6 | 71.6 |
| South Africa | 4753 (32.31) | 28.6 | 1.2 | 9959 (67.69) | 169.0 | 4.1 |  | 1313 (25.11) | 7.9 | 0.32 | 3917 (74.89) | 65.5 | 1.3 |  | 27.62 | 38.76 |
| South Sudan | 544 (50.7) | 23.5 | 0.93 | 529 (49.3) | 82.4 | 2.0 |  | 300 (45.66) | 13.2 | 0.52 | 357 (54.34) | 55.8 | 1.4 |  | 56.17 | 67.72 |
| Spain | 8684 (25) | 62.1 | 2.5 | 26051 (75) | 252.6 | 6.3 |  | 696 (10.32) | 4.8 | 0.19 | 6051 (89.68) | 41.1 | 0.93 |  | 7.73 | 16.27 |
| Sri Lanka | 1011 (22.2) | 16.5 | 0.67 | 3544 (77.8) | 98.0 | 2.4 |  | 280 (14.06) | 4.6 | 0.19 | 1711 (85.94) | 45.1 | 1.1 |  | 27.88 | 46.02 |
| Sudan | 3580 (53.99) | 37.1 | 1.5 | 3051 (46.01) | 108.9 | 2.6 |  | 1321 (40.73) | 13.8 | 0.55 | 1922 (59.27) | 69.7 | 1.5 |  | 37.2 | 64 |
| Suriname | 57 (36.08) | 36.5 | 1.4 | 101 (63.92) | 142.6 | 3.8 |  | 11 (20.75) | 7.2 | 0.28 | 42 (79.25) | 54.6 | 1.2 |  | 19.73 | 38.29 |
| Sweden | 1254 (16.83) | 49.4 | 2.0 | 6198 (83.17) | 286.1 | 7.1 |  | 108 (7.04) | 4.2 | 0.17 | 1427 (92.96) | 48.9 | 1.1 |  | 8.5 | 17.09 |
| Switzerland | 1178 (17.74) | 50.1 | 2.0 | 5464 (82.26) | 264.3 | 6.5 |  | 86 (5.58) | 3.6 | 0.15 | 1455 (94.42) | 53.0 | 1.2 |  | 7.19 | 20.05 |
| Syrian Arab Republic | 1884 (45.83) | 34.8 | 1.4 | 2227 (54.17) | 145.5 | 3.6 |  | 598 (33.58) | 11.3 | 0.45 | 1183 (66.42) | 77.1 | 1.9 |  | 32.47 | 52.99 |
| Tajikistan | 480 (58.68) | 20.5 | 0.82 | 338 (41.32) | 47.0 | 1.2 |  | 119 (42.05) | 5.4 | 0.22 | 164 (57.95) | 23.8 | 0.60 |  | 26.34 | 50.64 |
| Tanzania, United Republic of | 1739 (38.63) | 14.5 | 0.58 | 2763 (61.37) | 90.1 | 2.3 |  | 770 (34.15) | 6.6 | 0.27 | 1485 (65.85) | 48.3 | 1.3 |  | 45.52 | 53.61 |
| Thailand | 6397 (29.58) | 30.7 | 1.2 | 15226 (70.42) | 111.8 | 2.8 |  | 1417 (18.65) | 6.7 | 0.27 | 6182 (81.35) | 42.7 | 1.0 |  | 21.82 | 38.19 |
| The Netherlands | 2877 (18.07) | 68.5 | 2.7 | 13042 (81.93) | 340.2 | 8.3 |  | 220 (6.73) | 5.2 | 0.21 | 3049 (93.27) | 60.0 | 1.4 |  | 7.59 | 17.64 |
| The Republic of the Gambia | 68 (67.33) | 12.7 | 0.49 | 33 (32.67) | 29.4 | 0.64 |  | 29 (58) | 5.6 | 0.21 | 21 (42) | 19.3 | 0.40 |  | 44.09 | 65.65 |
| Timor-Leste | 57 (43.51) | 21.7 | 0.86 | 74 (56.49) | 78.8 | 2.0 |  | 14 (28.57) | 5.9 | 0.24 | 35 (71.43) | 36.9 | 0.89 |  | 27.19 | 46.83 |
| Togo | 514 (52.83) | 27.5 | 1.1 | 459 (47.17) | 97.2 | 2.4 |  | 256 (48.85) | 13.8 | 0.55 | 268 (51.15) | 56.6 | 1.5 |  | 50.18 | 58.23 |
| Trinidad and Tobago | 171 (30) | 40.0 | 1.6 | 399 (70) | 172.0 | 4.1 |  | 40 (15.69) | 9.5 | 0.38 | 215 (84.31) | 87.3 | 1.9 |  | 23.75 | 50.76 |
| Tunisia | 1186 (42.06) | 33.1 | 1.3 | 1634 (57.94) | 99.7 | 2.5 |  | 242 (26.86) | 6.8 | 0.27 | 659 (73.14) | 37.6 | 0.85 |  | 20.54 | 37.71 |
| Türkiye | 9746 (38.6) | 40.1 | 1.6 | 15502 (61.4) | 135.7 | 3.4 |  | 1768 (24.02) | 7.2 | 0.29 | 5592 (75.98) | 44.9 | 1.1 |  | 17.96 | 33.09 |
| Turkmenistan | 494 (49.35) | 29.3 | 1.2 | 507 (50.65) | 84.0 | 2.1 |  | 171 (35.26) | 10.3 | 0.41 | 314 (64.74) | 52.7 | 1.3 |  | 35.15 | 62.74 |
| Uganda | 1549 (51.96) | 17.5 | 0.69 | 1432 (48.04) | 73.6 | 1.8 |  | 744 (47.69) | 8.7 | 0.35 | 816 (52.31) | 41.9 | 1.1 |  | 49.71 | 56.93 |
| Ukraine | 3867 (21.37) | 28.5 | 1.1 | 14229 (78.63) | 145.5 | 3.8 |  | 674 (10.19) | 4.9 | 0.20 | 5939 (89.81) | 53.8 | 1.4 |  | 17.19 | 36.98 |
| United Arab Emirates | 488 (42.81) | 24.1 | 0.97 | 652 (57.19) | 225.6 | 5.5 |  | 81 (32.14) | 4.4 | 0.18 | 171 (67.86) | 68.6 | 1.5 |  | 18.26 | 30.41 |
| United Kingdom | 10153 (17.28) | 56.8 | 2.3 | 48602 (82.72) | 331.1 | 8.0 |  | 1014 (8.36) | 5.7 | 0.23 | 11108 (91.64) | 56.1 | 1.2 |  | 10.04 | 16.94 |
| United States of America | 49513 (18.05) | 58.3 | 2.3 | 224858 (81.95) | 336.8 | 8.3 |  | 4250 (9.91) | 5.0 | 0.20 | 38650 (90.09) | 48.7 | 1.1 |  | 8.58 | 14.46 |
| Uruguay | 467 (21.37) | 50.1 | 2.0 | 1718 (78.63) | 252.6 | 6.2 |  | 93 (11.97) | 10.0 | 0.40 | 684 (88.03) | 83.5 | 2.0 |  | 19.96 | 33.06 |
| Uzbekistan | 2388 (47.55) | 24.8 | 0.99 | 2634 (52.45) | 78.3 | 2.0 |  | 724 (32.24) | 7.8 | 0.31 | 1522 (67.76) | 45.9 | 1.2 |  | 31.45 | 58.62 |
| Vanuatu | 21 (56.76) | 28.4 | 1.1 | 16 (43.24) | 78.3 | 2.0 |  | 6 (54.55) | 8.4 | 0.33 | 5 (45.45) | 23.8 | 0.41 |  | 29.58 | 30.4 |
| Venezuela | 2658 (32.02) | 33.5 | 1.3 | 5642 (67.98) | 153.5 | 3.8 |  | 609 (20.63) | 7.7 | 0.31 | 2343 (79.37) | 61.9 | 1.5 |  | 22.99 | 40.33 |
| Viet Nam | 7931 (32.3) | 26.9 | 1.1 | 16623 (67.7) | 123.8 | 3.1 |  | 2013 (20.11) | 6.9 | 0.28 | 7995 (79.89) | 56.5 | 1.4 |  | 25.65 | 45.64 |
| Yemen | 1957 (68.14) | 29.0 | 1.1 | 915 (31.86) | 55.8 | 1.3 |  | 970 (63.48) | 14.5 | 0.58 | 558 (36.52) | 34.5 | 0.84 |  | 50 | 61.83 |
| Zambia | 459 (41.31) | 12.0 | 0.48 | 652 (58.69) | 82.2 | 2.0 |  | 170 (33.53) | 4.6 | 0.19 | 337 (66.47) | 42.6 | 1.0 |  | 38.33 | 51.82 |
| Zimbabwe | 731 (40.45) | 21.7 | 0.87 | 1076 (59.55) | 126.0 | 3.2 |  | 329 (35.96) | 10.0 | 0.40 | 586 (64.04) | 68.5 | 1.7 |  | 46.08 | 54.37 |

Note: ASIR=age-standardized incidence rate; ASMR= age-standardized mortality rate.

**Supplementary Table S3.** Projected number of cases, ASIR, ASMR, cumulative risk, and mortality-to-incidence ratio for premenopausal and postmenopausal breast cancer in 2050, by country and territory

|  | Incidence | | | |  | Mortality | | | |
| --- | --- | --- | --- | --- | --- | --- | --- | --- | --- |
|  | Premenopausal (age <50 years) | | Postmenopausal (age ≥50 years) | |  | Premenopausal (age <50 years) | | Postmenopausal (age ≥50 years) | |
|  | Cases (% of total cases) | Percentage change in number of cases (%) | Cases (% of total cases) | Percentage change in number of cases (%) |  | Cases (% of total cases) | Percentage change in number of cases (%) | Cases (% of total cases) | Percentage change in number of cases (%) |
| Afghanistan | 4817 (50.03) | 148.04 | 4812 (49.97) | 200.19 |  | 2044 (37.01) | 156.78 | 3479 (62.99) | 198.88 |
| Albania | 691 (24.74) | -20.61 | 797 (75.26) | 15.34 |  | 28 (4.54) | -12.5 | 589 (95.46) | 57.07 |
| Algeria | 6990 (38.1) | 16.79 | 14430 (61.9) | 106.44 |  | 2230 (24.2) | 16.33 | 6983 (75.8) | 134.72 |
| Angola | 1388 (46.06) | 153.77 | 4487 (53.94) | 223.27 |  | 1540 (39.43) | 155.39 | 2366 (60.57) | 224.11 |
| Argentina | 15340 (21.92) | 7.28 | 24036 (78.08) | 56.69 |  | 1162 (11.31) | 9.21 | 9114 (88.69) | 69.66 |
| Armenia | 834 (9.86) | -32.96 | 1097 (90.14) | 31.53 |  | 30 (4.78) | -31.82 | 597 (95.22) | 45.97 |
| Australia | 17630 (15.09) | 15.23 | 27894 (84.91) | 58.22 |  | 386 (6.46) | 15.92 | 5593 (93.54) | 82.78 |
| Austria | 4787 (14.53) | -17.64 | 5796 (85.47) | 21.08 |  | 101 (4) | -17.21 | 2425 (96) | 45.47 |
| Azerbaijan | 1340 (26.94) | -7.72 | 2140 (73.06) | 59.7 |  | 215 (17.93) | -5.7 | 984 (82.07) | 73.85 |
| Bahamas | 112 (22.01) | 13.33 | 241 (77.99) | 115.18 |  | 21 (10.55) | 16.67 | 178 (89.45) | 157.97 |
| Bahrain | 209 (17.55) | 6.96 | 578 (82.45) | 176.56 |  | 24 (8.3) | 9.09 | 265 (91.7) | 258.11 |
| Bangladesh | 5969 (34.45) | 17.8 | 15724 (65.55) | 163.43 |  | 2981 (22.02) | 20.84 | 10554 (77.98) | 185.63 |
| Barbados | 107 (20.13) | -15.79 | 127 (79.87) | 18.69 |  | 11 (10.58) | -15.38 | 93 (89.42) | 24 |
| Belarus | 4131 (14.45) | -26.52 | 4578 (85.55) | 10.82 |  | 125 (8.03) | -26.9 | 1431 (91.97) | 17.97 |
| Belgium | 9182 (15.27) | -3.33 | 11121 (84.73) | 21.12 |  | 132 (4.09) | -2.94 | 3093 (95.91) | 41.36 |
| Belize | 58 (18.91) | 40.74 | 163 (81.09) | 181.03 |  | 9 (16.07) | 50 | 47 (83.93) | 213.33 |
| Benin | 767 (40.03) | 133.63 | 1967 (59.97) | 156.45 |  | 632 (34.65) | 134.07 | 1192 (65.35) | 156.9 |
| Bhutan | 8 (33.33) | 37.5 | 22 (66.67) | 175 |  | 4 (22.22) | 33.33 | 14 (77.78) | 180 |
| Bolivia (Plurinational State of) | 798 (47.92) | 51.65 | 1452 (52.08) | 81.95 |  | 264 (31.62) | 58.08 | 571 (68.38) | 64.55 |
| Bosnia Herzegovina | 1197 (17.55) | -27.49 | 1165 (82.45) | -2.67 |  | 37 (6.06) | -27.45 | 574 (93.94) | 11.89 |
| Botswana | 143 (32.71) | 52.63 | 358 (67.29) | 150.35 |  | 53 (27.32) | 51.43 | 141 (72.68) | 151.79 |
| Brazil | 67437 (17.11) | -8.3 | 121253 (82.89) | 79.8 |  | 4396 (11.41) | -7.18 | 34119 (88.59) | 95.49 |
| Brunei Darussalam | 80 (23.3) | -5.88 | 158 (76.7) | 97.5 |  | 8 (10.39) | 0 | 69 (89.61) | 155.56 |
| Bulgaria | 2818 (14.7) | -41.76 | 2500 (85.3) | -11.28 |  | 76 (5.65) | -42.42 | 1270 (94.35) | 0.24 |
| Burkina Faso | 687 (46.33) | 147.3 | 1962 (53.67) | 185.59 |  | 907 (41.1) | 148.49 | 1300 (58.9) | 186.98 |
| Burundi | 465 (41.11) | 159.75 | 1470 (58.89) | 216.13 |  | 548 (36.22) | 166.02 | 965 (63.78) | 216.39 |
| Cambodia | 1145 (33.63) | 27.71 | 2437 (66.37) | 112.84 |  | 427 (23.32) | 30.18 | 1404 (76.68) | 138.37 |
| Cameroon | 1985 (46.48) | 120.7 | 5647 (53.52) | 184.48 |  | 2380 (40.87) | 120.57 | 3443 (59.13) | 185.49 |
| Canada | 26725 (13.5) | 14.28 | 37315 (86.5) | 39.63 |  | 630 (5.07) | 14.34 | 11785 (94.93) | 87.78 |
| Cape Verde | 32 (36.72) | 34.29 | 81 (63.28) | 153.13 |  | 9 (14.75) | 28.57 | 52 (85.25) | 225 |
| Central African Republic | 276 (60.72) | 201.34 | 581 (39.28) | 110.51 |  | 527 (55.65) | 201.14 | 420 (44.35) | 108.96 |
| Chad | 207 (70.87) | 163.74 | 580 (29.13) | 180.19 |  | 798 (66.56) | 164.24 | 401 (33.44) | 178.47 |
| Chile | 4152 (15.84) | -11.9 | 6964 (84.16) | 67.73 |  | 222 (7.02) | -10.12 | 2940 (92.98) | 92.41 |
| China | 245134 (21.9) | -24.17 | 302833 (78.1) | 23.54 |  | 9712 (8.74) | -24.27 | 101419 (91.26) | 63.16 |
| Colombia | 11352 (20.51) | -2 | 21501 (79.49) | 89.4 |  | 997 (11.27) | 2.05 | 7853 (88.73) | 108.03 |
| Comoros | 37 (30.99) | 57.14 | 98 (69.01) | 164.86 |  | 19 (24.36) | 58.33 | 59 (75.64) | 168.18 |
| Congo, Democratic Republic of | 3940 (47.28) | 170.31 | 10365 (52.72) | 163.07 |  | 4779 (42.25) | 170.31 | 6531 (57.75) | 162.71 |
| Congo, Republic of | 286 (42.58) | 109.43 | 689 (57.42) | 140.91 |  | 226 (40.57) | 111.21 | 331 (59.43) | 147.01 |
| Costa Rica | 1005 (15.61) | 0.88 | 1849 (84.39) | 83.98 |  | 67 (8) | 1.52 | 770 (92) | 115.69 |
| Côte d'Ivoire | 1660 (52.07) | 130.78 | 4687 (47.93) | 182.35 |  | 2546 (47.58) | 130.83 | 2805 (52.42) | 183.62 |
| Croatia | 2551 (13.42) | -32.68 | 2420 (86.58) | -5.14 |  | 34 (5.69) | -32 | 564 (94.31) | 0.36 |
| Cuba | 4412 (9.67) | -21.34 | 5477 (90.33) | 24.14 |  | 113 (4.41) | -20.98 | 2448 (95.59) | 49.63 |
| Cyprus | 752 (15.89) | -10.07 | 1276 (84.11) | 69.68 |  | 15 (3.33) | -6.25 | 436 (96.67) | 96.4 |
| Czechia | 6171 (15.24) | -25.24 | 6489 (84.76) | 5.15 |  | 90 (4.57) | -28 | 1879 (95.43) | 18.62 |
| Denmark | 4453 (14.49) | 11.41 | 5301 (85.51) | 19.04 |  | 66 (4.33) | 11.86 | 1460 (95.67) | 38.39 |
| Djibouti | 81 (42.66) | 55.56 | 207 (57.34) | 155.56 |  | 79 (37.26) | 58 | 133 (62.74) | 155.77 |
| Dominican Republic | 1906 (29.74) | 18.18 | 3733 (70.26) | 95.86 |  | 503 (18.65) | 21.5 | 2194 (81.35) | 110.56 |
| Ecuador | 2532 (23.65) | 24.95 | 5530 (76.35) | 118.4 |  | 327 (13.42) | 29.76 | 2110 (86.58) | 133.92 |
| Egypt | 14064 (39) | 60.23 | 32035 (61) | 127.78 |  | 5596 (27.2) | 60.57 | 14975 (72.8) | 145.05 |
| El Salvador | 1095 (21.41) | -0.39 | 1890 (78.59) | 72.6 |  | 90 (16.98) | 2.27 | 440 (83.02) | 80.33 |
| Equatorial Guinea | 64 (51.12) | 150 | 263 (48.88) | 310.94 |  | 132 (47.65) | 153.85 | 145 (52.35) | 314.29 |
| Eritrea | 225 (51.86) | 111.55 | 544 (48.14) | 141.78 |  | 300 (45.18) | 114.29 | 364 (54.82) | 141.06 |
| Estonia | 706 (13.58) | -27.15 | 700 (86.42) | -0.85 |  | 12 (4.24) | -25 | 271 (95.76) | 7.97 |
| Eswatini | 58 (33.61) | 70.83 | 162 (66.39) | 179.31 |  | 33 (31.13) | 73.68 | 73 (68.87) | 160.71 |
| Ethiopia | 7370 (49.56) | 124.74 | 21805 (50.44) | 195.86 |  | 11167 (44.47) | 127.53 | 13942 (55.53) | 195.51 |
| Fiji | 166 (37.76) | 31.71 | 267 (62.24) | 60.84 |  | 64 (21.4) | 33.33 | 235 (78.6) | 75.37 |
| Finland | 4732 (11.43) | 2.63 | 5135 (88.57) | 8.52 |  | 58 (5.64) | 3.57 | 970 (94.36) | 17.86 |
| France (metropolitan) | 52950 (16.65) | -1.2 | 62853 (83.35) | 18.7 |  | 1081 (5.38) | -0.55 | 19014 (94.62) | 39.28 |
| France, Guadeloupe | 181 (23.5) | 0 | 179 (76.5) | -1.1 |  | 11 (12.09) | 0 | 80 (87.91) | 29.03 |
| France, La Réunion | 276 (24.03) | 22.64 | 411 (75.97) | 48.91 |  | 27 (17.53) | 22.73 | 127 (82.47) | 86.76 |
| France, Martinique | 232 (18.7) | -12.5 | 213 (81.3) | -8.19 |  | 9 (10.59) | -10 | 76 (89.41) | 18.75 |
| French Guyana | 57 (24.74) | 65.52 | 146 (75.26) | 156.14 |  | 10 (14.08) | 66.67 | 61 (85.92) | 238.89 |
| French Polynesia | 93 (16.79) | 7.14 | 223 (83.21) | 139.78 |  | 11 (10.19) | 10 | 97 (89.81) | 177.14 |
| Gabon | 139 (45.77) | 76.63 | 385 (54.23) | 176.98 |  | 99 (38.22) | 76.79 | 160 (61.78) | 180.7 |
| Gaza Strip and West Bank | 455 (34.54) | 102.41 | 1431 (65.46) | 214.51 |  | 242 (25.42) | 106.84 | 710 (74.58) | 228.7 |
| Georgia | 1374 (15.62) | -29.49 | 1356 (84.38) | -1.31 |  | 65 (7.34) | -31.58 | 820 (92.66) | 7.05 |
| Germany | 64652 (9.78) | -15.52 | 72984 (90.22) | 12.89 |  | 1015 (3.98) | -15.49 | 24514 (96.02) | 26.36 |
| Ghana | 2427 (45.01) | 89.11 | 6004 (54.99) | 147.38 |  | 2166 (41.16) | 89.17 | 3096 (58.84) | 152.94 |
| Greece | 7356 (12.8) | -26.79 | 8135 (87.2) | 10.59 |  | 113 (3.72) | -27.56 | 2926 (96.28) | 28.62 |
| Guam | 42 (15.29) | 18.18 | 72 (84.71) | 71.43 |  | 2 (4) | 0 | 48 (96) | 118.18 |
| Guatemala | 1321 (30.52) | 60.02 | 3362 (69.48) | 154.5 |  | 276 (22.73) | 66.27 | 938 (77.27) | 156.28 |
| Guinea | 389 (42.81) | 122.94 | 974 (57.19) | 150.39 |  | 362 (38.63) | 129.11 | 575 (61.37) | 150 |
| Guinea-Bissau | 89 (44.91) | 109.28 | 249 (55.09) | 179.78 |  | 104 (38.24) | 112.24 | 168 (61.76) | 180 |
| Guyana | 163 (21.39) | 13.85 | 272 (78.61) | 66.87 |  | 20 (18.87) | 17.65 | 86 (81.13) | 72 |
| Haiti | 767 (34.45) | 39.8 | 1564 (65.55) | 103.91 |  | 311 (22.83) | 44.65 | 1051 (77.17) | 94.99 |
| Honduras | 719 (29.16) | 58.57 | 1888 (70.84) | 162.59 |  | 313 (28.15) | 57.29 | 799 (71.85) | 161.97 |
| Hungary | 6349 (12.27) | -30.93 | 6640 (87.73) | 4.58 |  | 115 (4.68) | -31.95 | 2340 (95.32) | 13.15 |
| Iceland | 167 (13.67) | 7.89 | 259 (86.33) | 55.09 |  | 8 (6.96) | 14.29 | 107 (93.04) | 84.48 |
| India | 123667 (24.8) | 24.32 | 257449 (75.2) | 108.18 |  | 30480 (15.65) | 26.61 | 164248 (84.35) | 121.23 |
| Indonesia | 38878 (31.23) | 8.2 | 65217 (68.77) | 67.75 |  | 7477 (20.56) | 8.71 | 28885 (79.44) | 83.83 |
| Iran, Islamic Republic of | 6868 (31.9) | -13.59 | 15892 (68.1) | 131.39 |  | 1550 (13.19) | -13.12 | 10199 (86.81) | 189.83 |
| Iraq | 4546 (37.5) | 104.63 | 13917 (62.5) | 206.14 |  | 2619 (27.82) | 105.73 | 6794 (72.18) | 223.68 |
| Ireland | 2820 (15.57) | -5.76 | 4435 (84.43) | 57.27 |  | 101 (6.17) | -6.48 | 1536 (93.83) | 98.19 |
| Israel | 3510 (21.07) | 41.79 | 5886 (78.93) | 67.69 |  | 213 (9.78) | 41.06 | 1966 (90.22) | 86.17 |
| Italy | 47181 (12.35) | -29.67 | 51391 (87.65) | 8.92 |  | 652 (3.29) | -30.56 | 19142 (96.71) | 31.87 |
| Jamaica | 803 (23.47) | -24.05 | 1298 (76.53) | 61.64 |  | 148 (15.1) | -18.23 | 832 (84.9) | 65.41 |
| Japan | 72440 (16.48) | -31.97 | 67144 (83.52) | -7.31 |  | 950 (5.37) | -33.1 | 16726 (94.63) | 3.13 |
| Jordan | 1339 (35.38) | 57.27 | 3616 (64.62) | 170.05 |  | 500 (25.97) | 58.23 | 1425 (74.03) | 193.21 |
| Kazakhstan | 3413 (21.05) | 15.47 | 5011 (78.95) | 46.82 |  | 347 (15.15) | 14.52 | 1944 (84.85) | 52.95 |
| Kenya | 4096 (34.45) | 91.45 | 11465 (65.55) | 179.91 |  | 2627 (31.38) | 93.16 | 5744 (68.62) | 181.84 |
| Korea, Democratic People's Republic of | 3866 (28.52) | -11.27 | 4618 (71.48) | 19.45 |  | 590 (30.24) | -11.41 | 1361 (69.76) | 20.44 |
| Korea, Republic of | 15066 (27.3) | -38.44 | 15909 (72.7) | 5.6 |  | 377 (9.82) | -37.38 | 3462 (90.18) | 49.81 |
| Kuwait | 540 (12.31) | -17.05 | 2080 (87.69) | 285.19 |  | 57 (5.21) | -22.97 | 1038 (94.79) | 473.48 |
| Kyrgyzstan | 509 (32.87) | 39.07 | 974 (67.13) | 91.36 |  | 90 (17.86) | 40.63 | 414 (82.14) | 100.97 |
| Lao People's Democratic Republic | 513 (36.49) | 42.88 | 1293 (63.51) | 152.05 |  | 201 (23.45) | 48.89 | 656 (76.55) | 163.45 |
| Latvia | 1027 (15.64) | -34.32 | 836 (84.36) | -18.6 |  | 20 (5.36) | -37.5 | 353 (94.64) | -11.31 |
| Lebanon | 1352 (24.49) | -34.41 | 1634 (75.51) | 20.86 |  | 141 (14.58) | -35.02 | 826 (85.42) | 46.45 |
| Lesotho | 128 (31.85) | 57.63 | 199 (68.15) | 55.47 |  | 38 (25.5) | 58.33 | 111 (74.5) | 54.17 |
| Liberia | 297 (50.26) | 111.08 | 679 (49.74) | 128.62 |  | 351 (45.17) | 112.73 | 426 (54.83) | 129.03 |
| Libya | 521 (37.9) | 9.26 | 1160 (62.1) | 122.65 |  | 191 (20) | 9.77 | 764 (80) | 167.13 |
| Lithuania | 1421 (14.75) | -31.54 | 1179 (85.25) | -17.03 |  | 30 (6.51) | -33.33 | 431 (93.49) | -8.3 |
| Luxembourg | 450 (12.57) | 1.74 | 814 (87.43) | 80.89 |  | 10 (5) | 0 | 190 (95) | 100 |
| Madagascar | 1616 (36.22) | 116.44 | 4799 (63.78) | 196.97 |  | 1237 (30.68) | 119.72 | 2795 (69.32) | 198.93 |
| Malawi | 753 (45.49) | 147.97 | 2347 (54.51) | 211.69 |  | 977 (39.44) | 149.87 | 1500 (60.56) | 211.85 |
| Malaysia | 5053 (25.41) | 18.55 | 11538 (74.59) | 128.34 |  | 1163 (15.09) | 20.64 | 6546 (84.91) | 155.5 |
| Maldives | 43 (22.49) | -9.52 | 131 (77.51) | 204.65 |  | 7 (10.45) | -12.5 | 60 (89.55) | 233.33 |
| Mali | 1029 (54.07) | 170.83 | 2871 (45.93) | 179.01 |  | 1811 (48.85) | 170.7 | 1896 (51.15) | 179.23 |
| Malta | 314 (10.96) | -20.83 | 463 (89.04) | 47.45 |  | 6 (4.72) | -14.29 | 121 (95.28) | 63.51 |
| Mauritania | 293 (46.29) | 113.68 | 761 (53.71) | 159.73 |  | 317 (42.15) | 114.19 | 435 (57.85) | 160.48 |
| Mauritius | 405 (17.25) | -10.61 | 566 (82.75) | 39.75 |  | 33 (11.07) | -8.33 | 265 (88.93) | 53.18 |
| Mexico | 19411 (24.45) | 5.79 | 38022 (75.55) | 95.88 |  | 2197 (14.45) | 9.58 | 13002 (85.55) | 110.05 |
| Mongolia | 123 (31.41) | 15.38 | 262 (68.59) | 113.01 |  | 26 (24.07) | 18.18 | 82 (75.93) | 141.18 |
| Montenegro | 335 (14.22) | -18.52 | 398 (85.78) | 18.81 |  | 11 (5.39) | -21.43 | 193 (94.61) | 37.86 |
| Morocco | 7441 (31.92) | 11.48 | 12635 (68.08) | 69.8 |  | 1324 (18.95) | 11.82 | 5662 (81.05) | 97.97 |
| Mozambique | 1070 (46.62) | 142.94 | 2721 (53.38) | 154.3 |  | 1230 (40.96) | 143.08 | 1773 (59.04) | 152.92 |
| Myanmar | 4124 (35.2) | 3.15 | 6685 (64.8) | 62.1 |  | 1099 (22.01) | 4.57 | 3895 (77.99) | 75.45 |
| Namibia | 319 (33.72) | 61.11 | 741 (66.28) | 132.29 |  | 139 (29.76) | 61.63 | 328 (70.24) | 132.62 |
| Nepal | 1232 (30.33) | 23.56 | 2904 (69.67) | 135.71 |  | 439 (18.42) | 27.62 | 1944 (81.58) | 141.49 |
| New Caledonia | 124 (20.21) | 11.54 | 229 (79.79) | 84.68 |  | 10 (13.33) | 11.11 | 65 (86.67) | 150 |
| New Zealand | 3061 (16.81) | 15.99 | 4558 (83.19) | 48.91 |  | 112 (8.9) | 16.67 | 1147 (91.1) | 73 |
| Nicaragua | 842 (19.74) | 34.06 | 2000 (80.26) | 137.53 |  | 138 (17.92) | 33.98 | 632 (82.08) | 140.3 |
| Niger | 782 (65.56) | 212.55 | 2238 (34.44) | 186.19 |  | 2500 (61.29) | 212.11 | 1579 (38.71) | 185.53 |
| Nigeria | 18458 (40.51) | 115.98 | 43831 (59.49) | 137.46 |  | 13789 (36.68) | 116.77 | 23802 (63.32) | 138.71 |
| North Macedonia | 793 (17.8) | -27.39 | 1016 (82.2) | 28.12 |  | 39 (7.56) | -26.42 | 477 (92.44) | 46.32 |
| Norway | 3685 (15.2) | 14.97 | 5101 (84.8) | 38.43 |  | 60 (4.73) | 15.38 | 1208 (95.27) | 69.42 |
| Oman | 199 (42.32) | 36.29 | 650 (57.68) | 226.63 |  | 151 (33.33) | 36.04 | 302 (66.67) | 243.18 |
| Pakistan | 16037 (42.04) | 91.15 | 38572 (57.96) | 140.52 |  | 9606 (26.97) | 98.51 | 26010 (73.03) | 142.79 |
| Panama | 693 (25.27) | 22.73 | 1437 (74.73) | 107.36 |  | 78 (12.81) | 25.81 | 531 (87.19) | 125.96 |
| Papua New Guinea | 898 (36.57) | 84.71 | 2451 (63.43) | 172.94 |  | 642 (28.34) | 85.01 | 1623 (71.66) | 193.49 |
| Paraguay | 1213 (28.8) | 20.42 | 2551 (71.2) | 110.31 |  | 188 (15.69) | 27.89 | 1010 (84.31) | 119.09 |
| Peru | 4531 (30.47) | 16.93 | 8713 (69.53) | 92.3 |  | 629 (17.85) | 20.04 | 2894 (82.15) | 102.8 |
| Philippines | 21434 (28.18) | 49.2 | 44247 (71.82) | 106.43 |  | 3986 (16.54) | 51.33 | 20108 (83.46) | 118.02 |
| Poland | 19417 (12.46) | -33.27 | 23446 (87.54) | 20.75 |  | 481 (4.34) | -34.11 | 10607 (95.66) | 32.7 |
| Portugal | 6819 (17.35) | -27.96 | 7329 (82.65) | 7.48 |  | 151 (5.53) | -28.77 | 2580 (94.47) | 29.06 |
| Puerto Rico | 1568 (11.97) | -13.27 | 1876 (88.03) | 19.64 |  | 48 (7.22) | -12.73 | 617 (92.78) | 37.42 |
| Qatar | 109 (38.56) | 34.53 | 298 (61.44) | 173.39 |  | 38 (26.21) | 35.71 | 107 (73.79) | 214.71 |
| Republic of Moldova | 1209 (17.59) | -43.07 | 1096 (82.41) | -9.35 |  | 50 (8.22) | -43.18 | 558 (91.78) | -4.94 |
| Romania | 10130 (14.87) | -26.26 | 10785 (85.13) | 6.47 |  | 259 (5.91) | -27.86 | 4123 (94.09) | 17.2 |
| Russian Federation | 61376 (16.31) | -27.22 | 65229 (83.69) | 6.28 |  | 1967 (8.09) | -27.9 | 22339 (91.91) | 15.23 |
| Rwanda | 362 (46.15) | 100.46 | 1022 (53.85) | 182.32 |  | 407 (41.83) | 102.49 | 566 (58.17) | 183 |
| Saint Lucia | 35 (30.49) | -19.35 | 57 (69.51) | 62.86 |  | 6 (17.65) | -14.29 | 28 (82.35) | 75 |
| Samoa | 54 (25.34) | 76.19 | 109 (74.66) | 101.85 |  | 8 (16.67) | 60 | 40 (83.33) | 110.53 |
| Sao Tome and Principe | 7 (52.78) | 111.11 | 17 (47.22) | 142.86 |  | 7 (41.18) | 133.33 | 10 (58.82) | 150 |
| Saudi Arabia | 1539 (33.15) | 16.26 | 5248 (66.85) | 241 |  | 589 (22.04) | 16.17 | 2084 (77.96) | 286.64 |
| Senegal | 873 (44.39) | 109.54 | 2531 (55.61) | 189.92 |  | 957 (38.54) | 110.79 | 1526 (61.46) | 192.9 |
| Serbia | 4014 (11.56) | -48.47 | 3487 (88.44) | -13.13 |  | 80 (4.57) | -48.05 | 1671 (95.43) | -1.18 |
| Sierra Leone | 80 (54.69) | 116.13 | 222 (45.31) | 177.5 |  | 143 (49.31) | 120 | 147 (50.69) | 177.36 |
| Singapore | 2600 (15.07) | -24.36 | 4131 (84.93) | 58.88 |  | 89 (4.93) | -23.93 | 1715 (95.07) | 97.81 |
| Slovakia | 2969 (10.62) | -33.24 | 3822 (89.38) | 28.73 |  | 65 (3.7) | -34.34 | 1691 (96.3) | 48.07 |
| Slovenia | 1351 (13.84) | -23.3 | 1476 (86.16) | 9.25 |  | 22 (3.54) | -24.14 | 600 (96.46) | 36.05 |
| Solomon Islands | 62 (43.75) | 88.06 | 162 (56.25) | 161.29 |  | 37 (30.58) | 85 | 84 (69.42) | 170.97 |
| Somalia | 924 (51.27) | 186.02 | 2411 (48.73) | 160.93 |  | 1459 (45.94) | 188.34 | 1717 (54.06) | 160.55 |
| South Africa | 9959 (21.2) | 17.1 | 20685 (78.8) | 107.7 |  | 1537 (14.17) | 17.06 | 9311 (85.83) | 137.71 |
| South Sudan | 529 (53.94) | 115.07 | 999 (46.06) | 88.85 |  | 653 (49.02) | 117.67 | 679 (50.98) | 90.2 |
| Spain | 26051 (16.61) | -29.88 | 30564 (83.39) | 17.32 |  | 481 (5) | -30.89 | 9147 (95) | 51.17 |
| Sri Lanka | 3544 (15.07) | -7.42 | 5276 (84.93) | 48.87 |  | 260 (8.43) | -7.14 | 2823 (91.57) | 64.99 |
| Sudan | 3051 (49.51) | 115.28 | 7860 (50.49) | 157.62 |  | 2872 (36.35) | 117.41 | 5029 (63.65) | 161.65 |
| Suriname | 101 (29.51) | 26.32 | 172 (70.49) | 70.3 |  | 14 (13.73) | 27.27 | 88 (86.27) | 109.52 |
| Sweden | 6198 (15.46) | 15.63 | 7932 (84.54) | 27.98 |  | 125 (5.84) | 15.74 | 2017 (94.16) | 41.35 |
| Switzerland | 5464 (12.95) | -4.67 | 7550 (87.05) | 38.18 |  | 82 (3.34) | -4.65 | 2374 (96.66) | 63.16 |
| Syrian Arab Republic | 2227 (35.86) | 120.75 | 7439 (64.14) | 234.04 |  | 1383 (25.17) | 131.27 | 4112 (74.83) | 247.59 |
| Tajikistan | 338 (48.9) | 62.08 | 813 (51.1) | 140.53 |  | 197 (31.88) | 65.55 | 421 (68.12) | 156.71 |
| Tanzania, United Republic of | 2763 (33.18) | 151.58 | 8811 (66.82) | 218.89 |  | 1958 (29.37) | 154.29 | 4709 (70.63) | 217.1 |
| Thailand | 15226 (18.89) | -25.2 | 20542 (81.11) | 34.91 |  | 1059 (9.1) | -25.26 | 10577 (90.9) | 71.09 |
| The Netherlands | 13042 (16.32) | 1.04 | 14902 (83.68) | 14.26 |  | 224 (4.97) | 1.82 | 4283 (95.03) | 40.47 |
| The Republic of the Gambia | 33 (58.89) | 133.82 | 111 (41.11) | 236.36 |  | 68 (48.92) | 134.48 | 71 (51.08) | 238.1 |
| Timor-Leste | 74 (40.66) | 94.74 | 162 (59.34) | 118.92 |  | 30 (28.3) | 114.29 | 76 (71.7) | 117.14 |
| Togo | 459 (46.45) | 101.36 | 1193 (53.55) | 159.91 |  | 516 (42.47) | 101.56 | 699 (57.53) | 160.82 |
| Trinidad and Tobago | 399 (17.95) | -12.87 | 681 (82.05) | 70.68 |  | 35 (7.58) | -12.5 | 427 (92.42) | 98.6 |
| Tunisia | 1634 (28.51) | -0.51 | 2959 (71.49) | 81.09 |  | 240 (13.83) | -0.83 | 1495 (86.17) | 126.86 |
| Türkiye | 15502 (25.79) | -3.97 | 26932 (74.21) | 73.73 |  | 1703 (13.05) | -3.68 | 11347 (86.95) | 102.91 |
| Turkmenistan | 507 (38.08) | 28.34 | 1031 (61.92) | 103.35 |  | 221 (24.61) | 29.24 | 677 (75.39) | 115.61 |
| Uganda | 1432 (47.45) | 158.1 | 4427 (52.55) | 209.15 |  | 1960 (43.71) | 163.44 | 2524 (56.29) | 209.31 |
| Ukraine | 14229 (14.52) | -41.43 | 13334 (85.48) | -6.29 |  | 392 (6.02) | -41.84 | 6125 (93.98) | 3.13 |
| United Arab Emirates | 652 (16.65) | 10.86 | 2708 (83.35) | 315.34 |  | 93 (9.62) | 14.81 | 874 (90.38) | 411.11 |
| United Kingdom | 48602 (14.04) | -1.83 | 61039 (85.96) | 25.59 |  | 998 (5.78) | -1.58 | 16263 (94.22) | 46.41 |
| United States of America | 224858 (16.2) | 14.77 | 293865 (83.8) | 30.69 |  | 4926 (7.71) | 15.91 | 58963 (92.29) | 52.56 |
| Uruguay | 1718 (16.84) | -5.35 | 2183 (83.16) | 27.07 |  | 89 (8.99) | -4.3 | 901 (91.01) | 31.73 |
| Uzbekistan | 2634 (34.53) | 17.67 | 5328 (65.47) | 102.28 |  | 856 (20.89) | 18.23 | 3241 (79.11) | 112.94 |
| Vanuatu | 16 (47.62) | 90.48 | 44 (52.38) | 175 |  | 11 (44) | 83.33 | 14 (56) | 180 |
| Venezuela | 5642 (26.51) | 33.71 | 9851 (73.49) | 74.6 |  | 830 (15.89) | 36.29 | 4395 (84.11) | 87.58 |
| Viet Nam | 16623 (19.31) | -11.5 | 29338 (80.69) | 76.49 |  | 1800 (10.23) | -10.58 | 15789 (89.77) | 97.49 |
| Yemen | 915 (57.71) | 119.37 | 3146 (42.29) | 243.83 |  | 2154 (52.86) | 122.06 | 1921 (47.14) | 244.27 |
| Zambia | 652 (33.05) | 147.28 | 2299 (66.95) | 252.61 |  | 428 (26.55) | 151.76 | 1184 (73.45) | 251.34 |
| Zimbabwe | 1076 (34.01) | 113.95 | 3034 (65.99) | 181.97 |  | 711 (30.15) | 116.11 | 1647 (69.85) | 181.06 |
